# Magnetic–Plasmonic Core–Shell Nanoparticles: Properties, Synthesis and Applications for Cancer Detection and Treatment

**DOI:** 10.3390/nano15040264

**Published:** 2025-02-10

**Authors:** Alberto Luis Rodriguez-Nieves, Suprava Shah, Mitchell L. Taylor, Madhusudhan Alle, Xiaohua Huang

**Affiliations:** Department of Chemistry, The University of Memphis, Memphis, TN 38152, USA; lrdrgez6@memphis.edu (A.L.R.-N.); sshah12@memphis.edu (S.S.); mltylor3@memphis.edu (M.L.T.); mralle@memphis.edu (M.A.)

**Keywords:** magnetic–plasmonic nanoparticles, core–shell, optical property, magnetic property, cancer detection, cancer treatment

## Abstract

Nanoparticles have been widely used in cancer diagnostics and treatment research due to their unique properties. Magnetic nanoparticles are popular in imaging techniques due to their ability to alter the magnetization field around them. Plasmonic nanoparticles are mainly applied in cancer treatments like photothermal therapy due to their ability to convert light into heat. While these nanoparticles are popular among their respective fields, magnetic–plasmonic core–shell nanoparticles (MPNPs) have gained popularity in recent years due to the combined magnetic and optical properties from the core and shell. MPNPs have stood out in cancer theranostics as a multimodal platform capable of serving as a contrast agent for imaging, a guidable drug carrier, and causing cellular ablation through photothermal energy conversion. In this review, we summarize the different properties of MPNPs and the most common synthesis approaches. We particularly discuss applications of MPNPs in cancer diagnosis and treatment based on different mechanisms using the magnetic and optical properties of the particles. Lastly, we look into current challenges they face for clinical applications and future perspectives using MPNPs for cancer detection and therapy.

## 1. Introduction

Cancer is a disease that affects more than 20 million people worldwide every year [1]. It is estimated that 1 in 5 people will develop cancer in their lifetime, while 1 in 9 men and 1 in 12 women will succumb to it [1]. Common approaches for clinical cancer diagnosis are imaging, tissue biopsy, and laboratory tests [2]. An imaging method, such as mammography, magnetic resonance imaging (MRI), positron emission tomography (PET), X-rays, ultrasound, and computed tomography (CT), is used to spot suspicious lesions in the body [3]. Then, a biopsy of the suspicious tissue confirms the malignancy of the disease and identify molecular markers for treatment selections. Laboratory tests, which detect abnormalities through biological markers in body fluids (blood, saliva, urine, etc.), including proteins, metabolic compounds, RNAs, DNAs, and circulating tumor cells (CTCs), are often used to assist the diagnosis or monitoring of treatment [4,5,6,7,8]. Despite the advancement of these mainstream approaches, each method has major limitations. The imaging methods can only detect a tumor when it reaches a certain size, about a few millimeters, depending on the method used and the location of the tumor. Tissue biopsy is inaccessible for some cancer types, impractical for repeated testing, and may cause further tumor dissemination [9,10,11,12,13]. Current laboratory tests with liquid biopsy are usually not sensitive enough to detect the early sign of metastasis.

The most common clinical methods to treat cancer are surgery, radiotherapy, chemotherapy, hormonal therapy, and immunotherapy, which are used alone or in combination. Each method has advantages and disadvantages. For example, surgery can remove whole tumor masses, but it is impractical for metastatic cancer. Chemotherapy kills cancer cells in the whole body, but it has severe side effects such as hair loss, nausea and vomiting, weight loss, and harm to the immune system because it kills both cancerous and healthy cells [14,15,16,17]. Immunotherapy uses antibodies, vaccines, viruses, checkpoint inhibitors, and other biological or biochemical interactions that can trigger an immune response to effectively combat cancerous cells [18,19,20,21]. However, like any other commonly used therapeutical approaches, immunotherapy can have various side effects such as pain, swelling, rash, and in some cases, fatal allergies and inflammatory reactions [22,23,24]. To increase patient survival, new therapeutic approaches are needed to more effectively treat cancer and prevent cancer metastasis.

Nanotechnology has been shown a promising tool to address the challenges in cancer detection and treatment that conventional methods cannot overcome [25,26,27,28,29,30,31]. This is partially due to the excellent sensitivity and selectivity of nanotechnology-based approaches. For example, Amrhein et al. reported a dual-imaging single-vesicle technology (DISVT) for surface protein profiling of individual extracellular vesicles (EVs) and early breast cancer detection using plasmonic gold nanoparticles (AuNPs) in conjunction with a direct capture method [32]. They compared early-stage BC patients and stage III BC patients against healthy donors, observing a significant difference in the fraction of HER2-positive plasma EVs. In contrast, the ELISA method was unable to detect early-stage BC patients. Compared to traditional approaches, the DISVT has several major advantages, including low sample consumption (<10 µL of 10–100 diluted plasma per assay), the detection of EVs down to the size of 40 nm, analysis of individual EVs, and the ability to differentiate between healthy donors and early BC patients.

Many types of nanomaterials have multiple properties, allowing them to perform multiple tasks simultaneously. The surface of these NPs can be modified with antibodies, drugs, and other biological molecules for target-specific detection and therapies [33]. One of the most commonly used materials is Gold (Au). AuNPs have been widely used in the biomedical field due to their unique localized surface plasmonic resonance (LSPR) property, ease of synthesis, facile surface modification, biocompatibility, low toxicity, and chemical inertness [34,35,36]. Particularly, they exhibit intriguing size and shape-dependent LSPR properties and biomedical functions. Adnan et al. compared spherical, rod, and star-shaped AuNPs for photothermal therapy (PTT) and drug delivery. They found that anisotropic (star and rod-shaped) AuNPs are better than their spherical counterparts regarding drug release and therapeutic efficacy in PTT.

Due to their tunable optical properties, AuNPs have also demonstrated potential for MRI, CT, photoacoustic, and X-ray cancer imaging. Similar to other types of NPs, AuNPs exhibit enhanced permeability and retention (EPR) in tumors after intravenous administration. In other words, they can accumulate at the tumor site and deliver drugs directly to the tumor, allowing for selective chemotherapy or localized tumor elimination with PTT [37,38]. For example, Cheng et al. developed a light-triggered assembly of 20 nm AuNPs for PTT and photoacoustic imaging [39]. In this work, they demonstrated the ability of the NPs to convert absorbed light into heat, which can be used to selectively destroy cancer cells with NIR irradiation at 808 nm while simultaneously providing photoacoustic imaging to detect the tumor.

Magnetic nanoparticles (MNPs) are another major class of nanoplatforms for cancer diagnosis and treatment. MNPs interact with external magnetic fields (MF), aligning their magnetic moments and rotating them in the direction of the applied field [40,41]. This reduces the relaxation time of the protons on the surrounding tissue of the MNPs in MRI and thus produces dark or brighter spots, providing a higher resolution image than traditional MRI [42,43,44]. Furthermore, MNPs have been used as enrichment tools for various biomedical applications. They can isolate and enrich CTCs for downstream molecular analysis via molecular targeting of surface protein markers such as epithelial cell adhesion molecules (EpCAM) [45,46,47]. Magnetic nanocarriers have also been widely used for passive and active tumor targeting and delivering cancer drugs in the tumor tissue for cancer therapy [48,49,50].

Magnetic–plasmonic nanoparticles (MPNPs) have become a topic of research in biomedicine due to their multimodal therapeutic and diagnostic abilities. By combining a magnetic core and a plasmonic shell, MPNPs will exhibit both magnetic and optical properties without the need to introduce them as individual entities. Of the existing material combinations, Fe-based core and Au shell are the most popular formulations due to their ease of synthesis, good stability, and biocompatibility [51,52,53,54]. Additionally, MPNP core size, shell thickness, and shape can be tuned according to their application needs. Song et al. conducted an extensive study on structure properties using Cobalt as the core and Au as the shell [55]. In their studies, they noticed that increasing shell thickness to 1.9 nm led to an increase in the coercivity at 10 K of the magnetic core. Inversely, the coercivity at 300 K decreased due to the partial loss of interparticle interaction. This suggests a correlation between the temperature and the interaction of the thermal energy fluctuation and the pinning effect of the Co-Au core–shell NPs.

In this review, we start with a discussion on the magnetic and optical properties of MPNPs based on both computational and experimental studies. This is followed by the major methods to synthesize and surface functionalization. Then, we summarize and discuss how MPNPs were used in cancer detection and then treatment based on the dual magnetic/optical properties of the MPNPs. Particularly, we emphasize the multimodal ability of the MPNPs in detection or treatment or both detection and treatment as a theragnostic agent. Lastly, we discuss some of the current limitations of MPNPs for clinical applications as well as future opportunities that are needed for further improvements and optimization.

## 2. Structural and Functional Properties

Because MPNPs have combined properties from the magnetic core and plasmonic shell, we therefore first discuss the properties of the core and shell materials as individual NPs. Then, we summarize the combined properties of MPNPs and the impact of the structure of core–shell MPNPs.

### 2.1. Magnetic Properties

**Figure 1:**
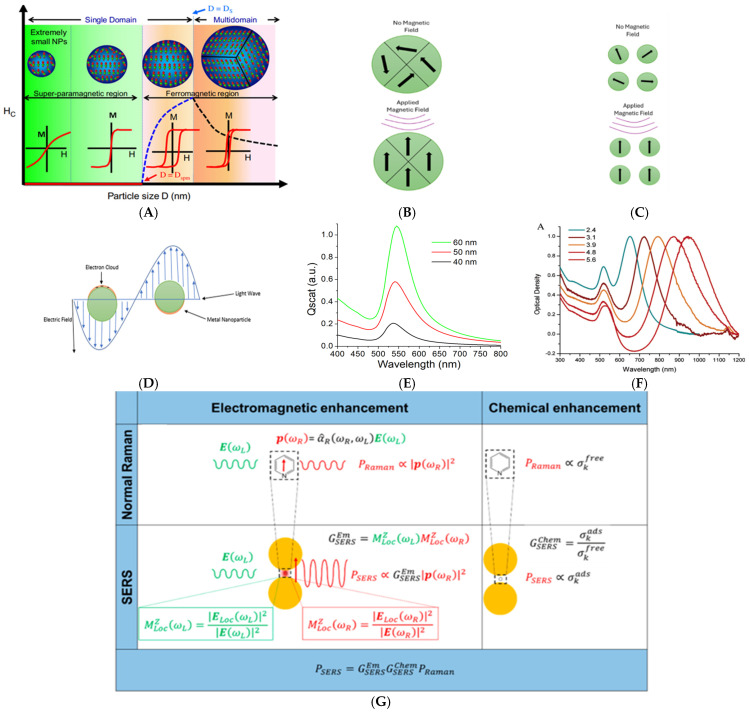
(**A**) Schematic of the size-dependent magnetic property for superparamagnetic NPs (**left**) and multidomain ferromagnetic NPs (**right**). Reprinted from ref. [61]. Copyright© 2018, with permission from Elsevier. (**B**,**C**) Schematic of the interaction of magnetic moments and magnetic field for ferromagnetic NPs (**B**) and superparamagnetic NPs (**C**). (**D**) Schematic of LSPR of an AuNP. (**E**) LSPR spectrum of AuNPs with different sizes. (**F**) LSPR spectrum of AuNRs with different aspect ratios. Reprinted with permission from ref. [83]. Copyright (2018) American Chemical Society. (**G**) Schematic of the principle of SERS from a molecule with and without a nano substrate. Reprinted from ref. [109].

The crystallinity degree and material purity usually define the magnetic properties of MNPs [56,57]. MNPs are typically fabricated with Fe, Co, Ni, and metal oxide such as magnetite (Fe_3_O_4_) and maghemite (γ-Fe_2_O_3_). Their size usually ranges from 1 to 100 nm, and they can be synthesized in different shapes [58,59,60]. The size and shape of the NPs have a large effect on their magnetic moments (Figure 1A) [61]. Studies have shown that the Ms of iron oxide (IO) NPs decreased as the size of the NPs decreased [62]. Magnetic materials are divided into paramagnetic, ferromagnetic, ferrimagnetic, diamagnetic, and antiferromagnetic. Paramagnetic materials (lithium, magnesium, aluminum) are characterized by random alignment of the magnetic moments in the absence of a magnetic field. Still, once an external MF is applied, their magnetic moments will align in the field direction [63,64]. Ferromagnetic materials (Fe, Co, Ni) have magnetic moments regardless of the presence or absence of an external field due to the unpaired electrons (Figure 1B) [65,66]. Ferrimagnetism (Fe_3_O_4_ or γ-Fe_2_O_3_) shows opposite magnetic moments at different sublattices. Although they exhibit opposite directions, their antiparallel moments do not cancel out each other since they have different magnitudes [67,68]. Diamagnetic materials (Au, Ag, Cu) have weak interactions with an MF, and they usually repel external MF. This is due to the lack of unpaired electrons in their atoms, which results in a zero net magnetic moment [69]. Antiferromagnetic materials (CoO, NiO, CuCl_2_) are composed of two types of atoms occupying different lattice positions, resulting in the alignment of the magnetic moments in the opposite direction of the applied MF, hence having no net magnetic moment [70,71]. Both antiferromagnetic and ferrimagnetic materials behave similarly to ferromagnetic materials in the presence of an external MF.

IO NPs (magnetite or maghemite) have good magnetic properties and biocompatibility. When the size of the IO NPs is smaller than 100 nm, the particles tend to have a single magnetic domain, acting as a single super spin (Figure 1C). Hence, the magnetization of these NPs is the sum of all the individual magnetic moments of the individual atoms of the MNPs [72,73]. In order to retain their magnetism properties, the MNPs need to have zero coercivity and no hysteresis [74,75]. In other words, when an MF is applied, the material becomes magnetized up to the saturation point. Still, upon the removal of the MF, the NPs lose their magnetic interaction and do not retain any magnetic residues. This phenomenon is known as superparamagnetism [76,77,78].

### 2.2. Optical Properties

When light interacts with an object, the photons can lose energy to molecules of the object, which induces the excitation of the molecules from the ground vibrational state to an excited state, a phenomenon called Stoke Raman scattering. Photons can also gain energy from a molecule, which will cause the molecules to experience the opposite process, called anti-Stoke Raman scattering [79,80]. These inelastic scattered photons contain information on the vibrational modes of the molecule. Generally, Raman spectra will report Stoke bands because their intensity is far stronger than anti-Stoke bands [81]. Raman scattering is a weak phenomenon compared to Rayleigh scattering. To put it in perspective, only 1 in 10 million incident radiation undergoes spontaneous Raman scattering. For this reason, Raman spectrometers are equipped with a filter to remove Rayleigh scattering [79,82]. At room temperature, most molecules are located in the ground state, whereas anti-Stoke constituents have far less intensity than Stoke components. This difference in the energy level population is described by the Boltzmann equation

(1) $$\frac{N_{ex}}{N_{g}}=\frac{g_{ex}}{g_{g}}e^{\frac{-(E_{ex}-E_{g})}{kT}}$$

where N_ex_ and N_g_ are the number of molecules in the excited and ground states, E_ex_ and E_g_ are the energies of the excited and ground states, and g is the degeneracy level. Although Raman is a weak phenomenon, molecules placed in close proximity to a suitable nanostructure can greatly amplify their signal intensities. This local field enhancement can be quantified by resorting to the classical view of Raman scattering.

P(*ω_R_*) = *α*^*R*(*ω*_*R*_, *ω_L_*)E(*ω_L_*) (2)

where E(*ω*_*L*_) is the external electric field, *ω*_*L*_ is the laser angular frequency that induces a dipole P(*ω*_*R*_) in the molecule, *ω*_*R*_ is the Raman angular frequency, and *α*^*R*(*ω*_*R*_, *ω*_*L*_) is the Raman polarizability tensor of the molecule.

When plasmonic materials (Au, Ag, Cu, Al) are exposed to the electromagnetic (EM) field of light, the free electrons of the metal oscillate collectively at the interface between a negative and positive permittivity material. A resonance occurs when the frequency of the incident light matches the natural frequency of the collective oscillation of the metal. When the size of the material decreases to the nanometer scale, the resonant oscillation is confined on a particle surface, which is termed LSPR (Figure 1D). LSPR is a unique size- and shape-dependent optical property of plasmonic nanomaterials (Figure 1E,F) [83]. Due to LSPR, plasmonic NPs absorb and scatter light strongly, orders of magnitude stronger than light-absorbing and fluorescent molecules. The absorbed light can also be converted into heat, a phenomena called the photothermal effect [84,85].

The size, shape, and composition of the NPs can greatly affect the optical properties of plasmonic NPs [86,87,88,89,90]. For instance, a spherical AuNP exhibits strong LSPR in the green spectral region (500–570 nm), which is responsible for the brilliant red color of the NP solution [91]. On the other hand, an AgNP of the same shape and size reflects a yellow color due to its LSPR in the blue spectral region (450–495 nm) [92,93]. The shape of NPs can also largely impact the LSPR wavelength. Due to their unique oscillation, gold nanorods (AuNRs) exhibit a longitudinal and a transverse LSPR. The transverse peak is usually visible in the 520–530 nm wavelength, and the longitudinal varies depending on the aspect ratio of the AuNRs [94,95]. Nanotriangles and nanostars are other anisotropic AuNPs that have multiple LSPR peaks and enhanced local EM field due to the high curvature of the NPs [96,97]. An example is the Ag nanotriangles (NTs) that show vastly different LSPR when they were synthesized at different seed concentrations (0.01, 0.1, 1 mL) [98]. The NTs synthesized with 0.01 mL seeds had their strong bands at 544 and 1234 nm due to in-plane dipole and quadrupole plasmonic resonance. It also showed an out-of-plane quadrupole at 339 nm. On the other hand, NTs prepared by 0.1 mL of seeds exhibited an in-dipole LSPR at 955 nm and an in-plane quadrupole at 478 nm, whereas its out-of-plane quadrupole was located at 340 nm. Lastly, the NTs prepared with 1 mL of seeds only exhibited an in-plane dipole and quadrupole at 528 and 428, respectively.

In 1974, a group of researchers led by Fleischmann first observed the phenomenon of surface enhancement Raman scattering (SERS) when they detected an increase in the Raman intensity of pyridine by a few orders of magnitude by adsorbing the molecule on a roughened Ag electrode [99]. Later, it was revealed that the signal enhancement was due to electromagnetic (EM) enhancement and chemical (CHEM) enhancement at the surface of the plasmonic nanomaterial [100,101,102,103,104,105,106]. The EM enhancement ($G_{SERS}^{EM})$ is believed to contribute 10^8^–10^10^ orders of magnitude to the signal intensity and can vary depending on the metal substrate and shape, making it independent from the molecule. Localized magnetic fields (hot spots) can amplify the EM up to 10 orders of magnitude. In order to achieve significant field enhancement, the molecules needs to be placed 1–10 nm in distance from the surface of the substrate [107,108]. The EM enhancement at the single molecule level can be calculated by adopting the |*E*|^4^ approximation, where

(3) $$G_{SERS}^{EM}\left(E^{4}\right)=M_{Loc}^{Z}\left(\omega_{L}\right)M_{Loc}^{Z}\left(\omega_{R}\right)={\left[\frac{E_{Loc}\left(\omega_{L}\right)}{E\left(\omega_{L}\right)}\right]}^{2}{\left[\frac{E_{Loc}\left(\omega_{R}\right)}{E\left(\omega_{R}\right)}\right]}^{2}$$

where $M_{Loc}^{Z}\left(\omega_{L}\right)\;and\;M_{Loc}^{Z}\left(\omega_{R}\right)$ refer to the field enhancements generated by a polarized laser along Z at the Raman and laser frequency. $E_{Loc}\left(\omega_{L}\right)$ is the local electric field generated by the laser, while $E_{Loc}\left(\omega_{R}\right)\;$ is the one generated by the Raman frequency (Figure 1G) [109]. The CHEM enhancement $\left(G_{SERS}^{CHEM}\right)$ originates from the polarization of a molecule arising from the physicochemical interaction between the molecule and the substrate. It is also dependent on the type of molecule. The CHEM enhancement contributes approximately 10^2^–10^4^ orders of magnitude to the SERS enhancement. It requires the molecule to be only a few angstroms from the substrate. The CHEM enhancement can be calculated by

(4) $$G_{SERS}^{CHEM}=\frac{\sigma_{k}^{ads}}{\sigma_{k}^{free}}$$

where $\sigma_{k}^{ads}$ and $\sigma_{k}^{free}$ represent the Raman cross-section of the *k*-th vibrational modes of the absorbed and free molecules. Throughout either physical (Van der Waals forces) or chemical absorption (chemical bonding) the electronic state and geometrical structure of the molecule undergoes modifications when interacting with the plasmonic surface, thus the Raman cross-section of the attached molecules will differ from those of the free molecules through their difference in the vibrational modes [109].

### 2.3. Tunable Properties of MPNPs

**Figure 2:**
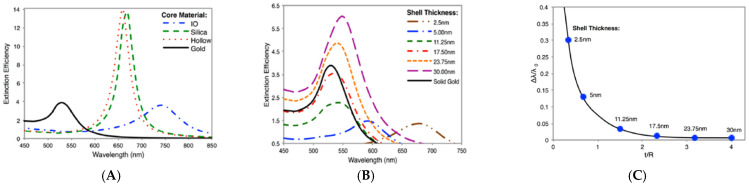

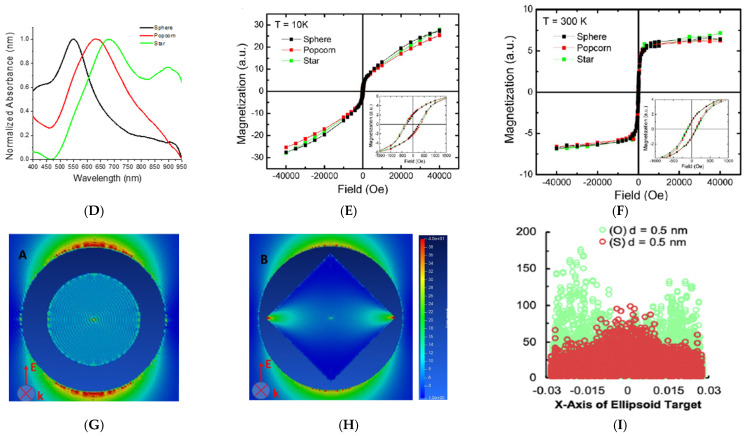
Optical and magnetic properties of IO-Au core–shell NPs. (**A**) Comparison of LSPR spectra of Au shell NPs with different core materials. (**B**) LSPR spectrum of IO-Au core–shell NPs with varying shell thickness. Core diameter = 15 nm. (**C**) Fractional shifts (Δλ/λ0) of the LSPR peak maximums of the IO-Au NPs from (**B**). Reprinted with permission from ref. [114]. Copyright (2014) American Chemical Society. (**D**) LSPR spectrum of IO-Au core–shell NPs with different shell geometry. (**E**,**F**) Magnetic property of IO-Au core–shell NPs with different shell geometry at temperature = 10 k (**E**) and at temperature 300 k (**F**). Reprinted with permission from ref. [131]. Copyright (2016) American Chemical Society. (**G**) |E2|/|E02| field maps for an IO-Au core–shell with a spherical core with 35 nm diameter and an overall 55 nm diameter at wavelength 660 nm. (**H**) |E2|/|E02| field maps for an IO-Au core–shell with a octahedral core with 35 nm edge length and overall 55 nm diameter at wavelength 660 nm. (**I**) Near-field enhancement factor for surface points on shell with spherical (diameter 35 nm) and octahedral core (edge length 35 nm) at wavelength 660 nm. Reprinted from ref. [132].

Compared to the individual NPs made of the core and shell materials, the advantages of core–shell NPs are the combined properties from the core and shell materials as well as the tunability of the properties by manipulating the structure of the hybrid NPs, including the core and shell sizes and geometry as well as the composition of the core and shell materials [110,111,112,113]. As shown in a computational study by Wang and co-authors with extended Mie theory, the dielectric property of the core largely impacts the LSPR of the core–shell NPs (Figure 2A) [114]. The LSPR of IO-Au core–shell NPs is red-shifted compared to solid AuNPs or Au shell NPs with a silica or hollow core. However, the intensity is comparable to that of solid AuNPs and much reduced than hollow Au and Si-Au core–shell NPs. In contrast, Co-Au core–shell NPs have very weak plasmon peaks, although Co has stronger magnetic properties than magnetite NPs. These optical differences are due to the differences in dielectric properties of the core, which are related to the refractive index of the material. IO NPs are nontransparent and have complex refractive indices. Silica, on the other hand, is a non-absorbing material and has only a real refractive index. Similarly, plasmonic metal shells like Au, Ag, Cu, and Pt have different dielectric properties and thus exhibit different LSPR wavelengths [115,116,117,118,119,120].

The LSPR of core–shell is also largely impacted by the shell thickness [121,122,123,124,125,126]. Wang and co-authors systematically investigated the effect of Au shell thickness on IO-Au core–shell NPs [114]. Their results show that a blue shift of the LSPR peak was observed with an increase in the Au-shell thickness when the core size is fixed (Figure 2B). However, as the Au-shell layer continues to grow, a red shift of the LSPR peak occurs. This threshold is around 17.5 nm for a 15 nm IO core. This effect follows a universal scaling when plotting the fractional shifts (Δλ/λ0) of the LSPR peak maximums against the ratio of the shell thickness to the radius of the core (t/R) (Figure 2C), a similar effect that has been observed with the Si-Au core–shell nanosystem [127]. The shape of the shell or core also affects the LSPR of the MPNPs. Not surprisingly, a change of the shell shape from sphere to rod led to the emergence of a longitudinal LSPR peak corresponding to the electron oscillations along the long axis of the rod [128,129,130]. A change of the Au shell from sphere to star resulted in an additional plasmon peak in the NIR region (Figure 2D) [131]. However, the geometry of the core and shell have negligible effects on the magnetic properties (Figure 2E,F). It is expected that the optical properties of IO–Au NSTs can be further tuned by changing the number and length of the Au tips. In such complex systems, plasmon coupling between the core and shell as well as among the tips also contributes to the presence of the multiple LSPR peaks. The geometry of the core–shell is also a major player for the near field. For example, a change of the IO core from sphere to octahedral induced an increase in the near field of IO-AuNPs, due to the presence of more hot spots around the anisotropic IO core (Figure 2G–I) [132].

While the production of isotropic spherical core–shell MPNPs is the most common synthesis approach due to its ease of synthesis, stability, and better distribution of functional groups in surface modifications, other groups have explored the use of anisotropic MPNPs due to the many possible advantages related to the shell geometry. In plasmonic nanomaterials, a non-spherical geometry can allow plasmon resonance tunning across a wider optical spectrum by changing the shape, aspect ratio, and size, while spheres are limited to only their size. Furthermore, anisotropic shapes promote the generation of electromagnetic hotspots at sharp edges and tips, greatly enhancing the local electromagnetic field (EMF), which can greatly boost SERS signaling and sensitivity. The formation of multi-plasmon modes (longitudinal and transverse) from shapes such as stars and rods can better support multimodal approaches in diagnosis and therapy thanks to their multi-wavelength functionality. The transverse plasmon band is sensitive to changes in the local dielectric environment, which allows for better biosensing and refractive-index detection.

Wang et al. characterized the properties of magnetic hematite core–gold shell Nanorice to learn about its possible application as a highly sensitive sensor for chemicals and biological processes [133]. They reported that their core–shell structure longitudinal plasmon band was highly sensitively dependent on the surrounding dielectric media, reaching refractive index units (RIU) as high as 801 nm RIU^−^^1^. RIUs are obtained when the refractive index of a sample and its medium changes resulting in resonant mode shifts to a new wavelength [134,135]. In their work, they reported that their platform achieved larger SPR sensitivity than triangular nanoprisms, nanorods, spherical nanoshells, and nanocubes. This SPR sensitivity holds great potential for monitoring local environmental changes during chemical and biological processes [133].

Wilson et al. conducted experiments on the use of iron oxide core–gold shell nanopopcorns for immunomagnetic capturing and multiplex detection of circulating tumor cells [136]. Popcorn-shaped shells were chosen to improve SERS detection sensitivity as it is orders of magnitude stronger than their spherical counterparts due to stronger EF enhancement originating from the island formation along the (111) and (11-1) facets of the gold shell. This enhancement factor allows to reach high sensitivity enabling single-molecule detection. Their work allowed cancer cell isolation/enrichment, optical imaging, multiplex SERS detection for four cancer biomarkers (EpCAM, HER2, CD44, and IGF1R), as well as molecular profiling of various breast cancer cell lines. All of this was performed on a microfluidic device, increasing the degree of operational automation while facilitating single-cell analysis [136].

One of the fundamental aspects of plasmonic anisotropic nanomaterials is their ability to be tailored at the specific wavelength needed for its application. Tomikata et al. implemented magneto-plasmonic nanostars for image-guided and NIR-triggered drug delivery [137]. As the SPR of gold materials is dependent on different geometrical aspects, spherical shells of SPR are mainly located in visible regions below 600 nm. On the other hand, rod- and star-shaped NPs can be tuned to induce SPR red-shifting and reach the NIR region. In their work, achieving NIR SPR was necessary to trigger the release of tenofovir disoproxil fumarate (TDF), an antiretroviral drug. The core–shell NSTs were able to reach a drug-binding capacity of 23 ug TDF/mg NSTs after 1 h of reaction. After stimulating the NSTs through NIR exposure, drug release was observed after only 5 min. After 30 min of exposure, the drug released reached almost 3× the amount of that of 5 min. Furthermore, the NSTs were tested using MRI, MPI, and PAI for multimodal imaging. The results showed the T_2_ (transverse) relaxation rate to be 218 mM^−1^s^−1^, which was higher than that of the T_2_ clinical contrast agent Feridex (133 mM^−1^s^−1^). Higher relaxation times generate better image resolution [137].

**Figure 3:**
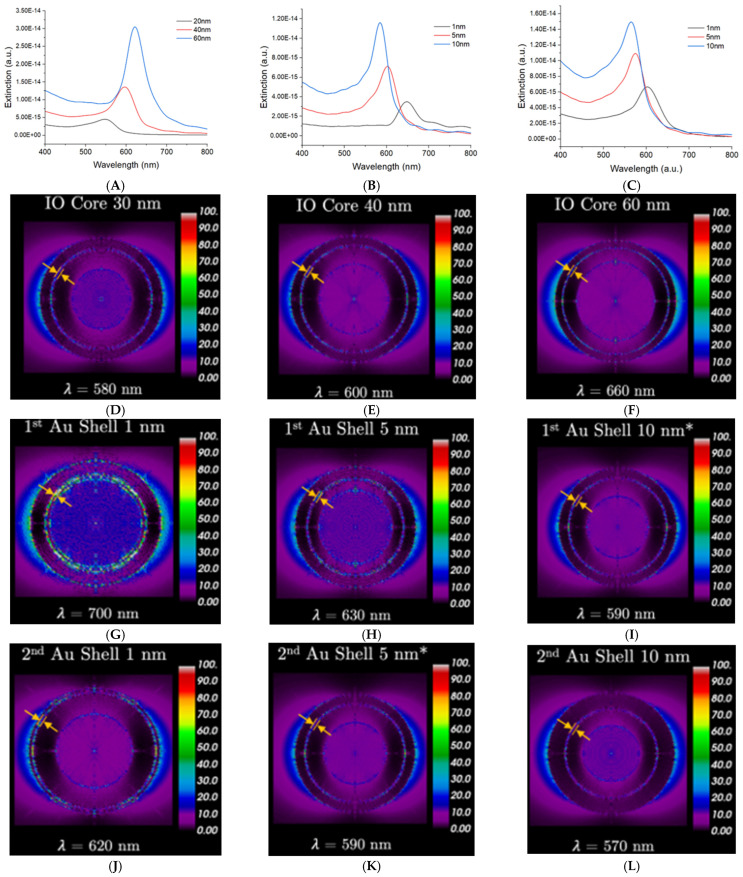
Optical properties of IO@Au@gap@Au NPs. Calculated extinction spectra with different IO core sizes (**A**), different thicknesses of the inner Au shell (**B**), and different thicknesses of the outer Au shell (**C**). Calculated electric field enhancement |E/E_0_|^2^ in the x-y cross-section taken from the center of the particle at a specified wavelength with varying inner and outer shell thicknesses (**D**–**L**). *: the structural parameters of IO@Au@gap@Au NPs that are not varied when other parameters are varied (IO core: 35 nm, first Au shell: 10 nm, gap: 1 nm, second Au shell: 5 nm). The arrow on each panel points out the gap between the first and second Au shell. Reprint with permission from ref. [138]. Copyright (2024) American Chemical Society.

Other complex cases involve the addition of another plasmonic shell as a means to create a gap-enhanced system. Rodriguez et al. conducted computational simulations by finite-difference time domain (FDTD) and discrete dipole approximation (DDA) to study the optical properties at different geometrical aspects of IO/Au/Au NPs [138]. When increasing the magnetic core size, the plasmon peak moves toward longer wavelengths (lower energies) (Figure 3A). They attributed this to the increase in the inner and outer shell size which is proportionally affected by the core size. Afterward, both the inner and outer shell thicknesses were changed to observe their effects on the NP LSPR. The results indicated a blueshift in the LSPR attributed to the system behaving as a solid gold sphere (Figure 3B,C). Furthermore, the peak intensity increases due to the dampening effect of the IO core on the inner decreasing. Upon varying different geometrical aspects of the core–shell-shell system, they observed that the extinction spectra only consisted of an individual peak rather than two belonging to both the inner and outer shell. The absence of a second peak was attributed to the dampening of the plasmonic response from the inner shell due to the presence of an IO core that caused the antibonding (nonbonding) peak to disappear. Thus, they concluded that the presence of a single peak was the effect of the bonding mode between the inner and outer gold shells. In addition to the LSPR, electrical field maps were generated to observe the electromagnetic field (EMF) enhancement. As the IO core size increases, the EMF increases (Figure 3D–F). Additionally, by decreasing the inner or outer shell thickness, they produced stronger EMF in the gap between the two gold shells (Figure 3G–L).

### 2.4. Effects of the Magnetic Core Size and Shape in the Plasmon Behavior

Magnetic cores can directly influence the dielectric constant around themselves based on different physical aspects. This leads to changes in the plasmon behavior of the shell due to the high sensitivity that plasmonic resonance has to changes in the dielectric field. The presence of a strong magnetic moment close to the plasmon modes of the shell can introduce various physical effects such as magneto-plasmonic coupling, which leads to hybrid resonance modes, enhanced nonlinear optical effects based on Kerr’s effect, as well as magnetic circular dichroism enhancement, which can allow for the plasmon resonance to be dynamically tuned by applying an external magnetic field [139,140,141,142,143]. In the hybridization theory, the plasmon response of a shell is related to the interaction of the plasmonic response of the core and the cavity [144]. Brullot et al. studied the implications of changing different aspects of the magnetic core–gold shell nanoparticles and the changes in the optical properties [145]. For instance, by varying the magnetic core diameter from 0 to 70 nm while keeping the total diameter size at a maximum of 70 nm, they observed a plasmon redshift reaching NIR at 60 nm with minimal influence in the Q_extinsion_. At shorter wavelengths, a resonant peak attributed to the quadrupolar interaction in the dielectric shell could be observed, while a dipolar plasmon band contributing to most of the plasmonic resonance was present at longer wavelengths. Brullot also studied the effects of a rod-shaped magnetic core–shell NP, which showed that by increasing the volume ratio of the V_core_/V_shell_ from 0 to 1, the LSPR redshift was attributed to the decrease in the gold as the V_core_ increased. Furthermore, increasing the aspect ratio of the core–shell caused the longitudinal plasmon band to redshift while also increasing its Q_extinsion_. This was expected as a higher aspect ratios increase the path length for electron oscillation along the longitudinal axis. Lastly, it was noticed that increasing the volume of the NRs while keeping other parameters constant had only a small effect on the plasmon band shifting, whereas its Q_extintion_ increased. This is due to the amount of material present in a single nanostructure [145].

Levin et al. conducted experiments by changing the core geometry while keeping the gold shell spherical to visualize what possible effects this could have on the structure’s optical properties [146]. Levin determined that in the case of a core composed of a high-permittivity material (iron oxide), there is no strong dependence on the core geometry and the wavelength shift. This is because the shape of the dielectric core has a minor influence on the extinction spectra or the antibonding dipolar nanoshell resonances. Similar tests were conducted on a low-permittivity (silica) core, observing the bonding dipolar nanoshell resonances where opposite trends were observed. This concluded that the dielectric permittivity of the core is the primary controlling factor in the plasmon resonance response whereas the shape plays a minor influence on it [146].

Other groups have studied the plasmon shell’s effects on the core’s magnetic properties, obtaining mixed results. For instance, Wang et al. studied a nickel (Ni) core–gold (Au) shell structure while alternating the metal ratios [147]. They found that the measure values of saturation magnetization (M_s_) increase as the magnetic core diameter increases. However, ratios where the magnetic core–plasmonic shell was Ni_50_:Au_50_ and Ni_60_:Au_40_, showed lower M_s_ than pure Ni NPs. This was because of a decrease in the magnetic core diameter and a high composition of the diamagnetic gold shell. Nonetheless, they observed that when the ratio reached Ni_80_:Au_20_, the M_s_ was found to be higher than pure Ni NPs. This was attributed to metallic electrons trapped in the Ni–Au interface due to the chemical potential gradient generated, which allowed the trapping of the gold electrons, which generated an orbital moment in the interface. SERS results indicated that at a ratio of Ni_50_:Au_50_, the enhancement factor (EF) reached 2.5 × 10^6^ or nine times higher than for gold-only (Au_100_) NPs, which was only 2.8 × 10^5^. They concluded that it was due to intense electromagnetic coupling between adjacent NPs as well as enhanced LSPR between the magnetic cores and the surface polarization of the Au shell for each NP [147].

Kwizera et al. reported that the magnetism of the core–shell structure can be greatly influenced and adjusted by altering factors such as core surface, which can be achieved by decreasing its size resulting in loss of magnetism, or by passivation of surface effect due to layering which is achievable by introducing a inorganic or organic layer (e.g., gold shell) resulting in distortion of the core magnetic moment due to interactions with the gold shell electrons [148]. The latter can cause a phenomenon called spin canting, which results in the core–shell NP having larger surface effect than pure IONPs due to structural distortion. Furthermore, increases in the shell thickness generally lead to a decrease in magnetic moments due to the mass contribution of the diamagnetic shell. However, in previous studies, they reported that increased shell thickness led to better magnetic properties of core–shell nanopopcorns at 10 K [131]. These NPs were capped with CTAB, which interacts and binds to the gold through Br-Au bonding while creating a positively charged CTA^+^ bilayer, leading to enhanced electron mobility from to the Br-Au charge transfer, which could induce magnetism of the gold shell [131].

## 3. Synthesis Methods

### 3.1. Direct Deposition Method

A common way to synthesize MPNPs is to synthesize magnetic core NPs first and then reduce metal precursors in the presence of the magnetic core NPs. The magnetic core NP surface catalyzes the reduction of metal ions, which makes metal deposits onto the core NPs to form core–shell NPs rather than solid metal NPs. Co-precipitation is the most common and widely used method for the synthesis of magnetic nanoparticles. This method is popular due to its simplicity, cost-effectiveness, and high yield of nanoparticles with controlled size and magnetic properties [149,150]. The precipitation of iron ions is carried out in an aqueous solution (e.g., sodium hydroxide or ammonium hydroxide) containing ferric (Fe^3+^) and ferrous (Fe^2+^) salts as precursors under inert conditions [151,152]. Metal precursors such as chloroauric acid (HauCl_4_) or silver nitrate (AgNO_3_) are reduced onto the magnetic cores using a suitable reducing agent to achieve MPNPs. For example, Tamer et al. synthesized Fe_3_O_4_–Au core–shell NPs by the reduction of HAuCl_4_ with sodium borohydride under sonication. The resulting solution was dark red, indicating the formation of the Fe_3_O_4_–Au core–shell NPs. The mean hydrodynamic diameter of Fe_3_O_4_ and Fe_3_O_4_–Au NPs was 100 and 122 nm, respectively, according to dynamic light scattering (DLS) measurements [153]. Fe_3_O_4_@SiO_2_-Ag MPNPs with a magnetic Fe_3_O_4_ core, a silica (SiO_2_) coating, and a silver (Ag) plasmonic shell were synthesized by Chu et al. [154]. These are spherical NPs with rough surfaces with an average diameter of 150 nm. X-ray diffraction (XRD) demonstrated that the saturation magnetization of the NPs reduced as the Ag content increased, whereas the intensity of the Ag diffraction peaks increased. The presence of the nonmagnetic Ag shell caused a decrease in magnetism.

### 3.2. Seed-Mediated Growth Method

Seed-mediated growth method is one of the widely used techniques to synthesize core–shell structures in controllable sizes and shapes. MNPs serve as seeds, and the plasmonic shell is grown layer-by-layer in a controlled manner. In this method, a small amount of metal ions (e.g., Au^3+^, Ag^+^) are reduced to elementary forms and are deposited on the magnetic core, followed by further growth by adding an additional gold precursor and reducing agent [94,155]. The core–shell NP exhibits distinct core–shell morphology with a well-defined interface between the magnetic core and the plasmonic shell. This method allows precise control over the shell thickness and morphology, leading to highly uniform nanoparticles with tunable optical and magnetic properties [156]. Star-shaped magnetic–plasmonic Au@Fe_3_O_4_ nanoparticles were synthesized by Muzzi et al. via seeded-growth approaches. The Au@Fe_3_O_4_ nanostars had an average size of (60 ± 10) nm, comprising an Au core and a highly uniform Fe_3_O_4_ shell. The extinction spectrum of AuNPs reveals a plasmonic resonance at 530 nm, which redshifts at 640 nm when the magnetite shell surrounds gold seeds [157]. Wei et al. synthesized magnetic–plasmonic FePt-Au core–shell NPs by seeding gold acetate Au(ac)_3_ with FePt NPs. The synthetic FePt core NP had an average diameter of roughly 2.5 nm, while the FePt–Au core–shell NPs had a larger size of 6.5 nm. The selected area electron diffraction (SAED) pattern of a particle ensemble displays diffraction rings corresponding to both face-centered cubic (fcc) Au and fcc FePt, indicating the presence of FePt–Au core–shell nanoparticles. Similarly, the longer wavelength (redshift) of core–shell NP SPR band than pure NPs indicates gold absorption on the surface and FePt–Au core–shell NP formation [158]. Trang et al. synthesized FePt@Ag core–shell nanoparticles using OLA-capped FePt NPs as seeds to grow on Ag shells. The diameter was found to be 15.9 ± 1.5 nm. EDS elemental mapping showed the presence of Pt in the core and Ag as the shell. These NPs exhibited both superparamagnetic behavior and strong plasmonic properties due to the silver shell [159].

Core–shell synthesis involves the formation of NPs with one particle as the core and another as the shell [160]. It involves the deposition of magnetic seeds and their ability to interact with the metal shell (such as gold or silver). The cores are usually stabilized either by silica or polymers to form a plasmonic shell around it. In core–shell structures, the shell physically separates the “active core” from the surrounding medium, preventing photooxidation, environmental variations, and surface chemical changes [161].

**Figure 4:**
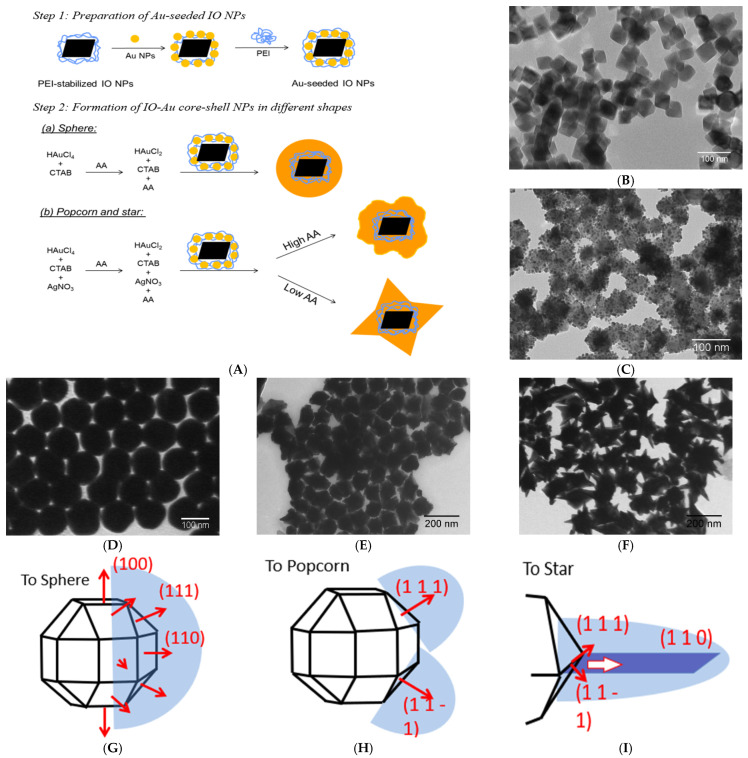
Size- and shape-controlled synthesis of IO-Au core–shell NPs. (**A**) Schematic of the synthesis of the iron oxide core-gold shell NPs in different shapes by changing ascorbic acid concentrations. (**B**) TEM image of the octahedral IO core. (**C**) TEM image of the octahedral IO core adsorbed with Au seeds. TEM images of IO-Au core–shell NPs with spherical shell and total size of 100 nm (**D**), popcorn shell and total size of 89 nm (**E**), and star shell and total size of 100 nm (**F**). Growth mechanism of a spherical shell (**G**), popcorn shell (**H**), and star shell (**I**). Reprint with permission from ref. [131]. Copyright (2016) American Chemical Society.

Kwizera et al. carried out the synthesis of IO–Au core–shell NPs in three different shapes (nanostars, nanopopcorn, and nanospheres) with the combination of precipitation and seed-mediated methods (Figure 4A) [131]. IO NP was prepared by the precipitation of FeSO_4_ in aqueous solution, leading to the formation of octahedral cores (Figure 4B). The presence of positive charges on the aqueous soluble IO NPs allows for the subsequent adsorption of negatively charged Au seeds via electrostatic interactions (Figure 4C). AuNP seed stabilized with polyethyleneimine (PEI) serves as the nucleation site to facilitate the growth of the Au shell. The amount of PEI on the IO NPs determines the surface density of the Au seed. The increased quantity of PEI results in the higher density of Au seeds. Furthermore, by changing the concentration of AgNO_3_ and ascorbic acid (AA), different shell shapes were achieved. AgNO_3_ is required to form anisotropic shells like stars and popcorn. In the absence of AgNO_3_, the (100), (110), and (111) faces of the gold seeds are readily available to react with the gold precursor, leading to the formation of spherical NPs (Figure 4D,G). When silver ions are present, they are reduced and deposited onto the surface of the gold seeds in the presence of AA. To achieve anisotropic growth, the ions must be deposited at different rates and crystal faces. By adding high concentrations of Ag^+^, they are able to attack the (110) and (100) surface of the gold seeds, thus blocking the absorption of gold precursor on those faces. This leaves the (111) face as the only readily available site for gold to react with, hence forming anisotropic-shaped NPs. AA helps control the kinetic deposition of gold into the faces of the seeds. When Au^+^ is quickly reduced, the gold precursor is deposited in the (111) facet, leading to a gold island growth along the (111), which is characteristic of nanopopcorns (Figure 4E,H). Inversely, as the reduction of the gold ion becomes slow, the gold precursor is deposited mainly at the ridge formed between the (111) and (11-1) planes, leading to the formation of sharp spikes (Figure 4F,I). This is due to the incoordination between the gold atoms at the ridge and the ones located at the center of the facet. This effect causes strong gold absorption while inducing a stronger electrical field at the ridge. Plasmonic redshifts for all three shapes could be observed as the thickness of the gold shell increased. Magnetization experiments were conducted to understand the effects of shell thickness on the magnetic core. At 10 K, the NPs presented a magnetization dependency for both ferromagnetic and paramagnetic modes. They believed the paramagnetic response belonged to the gold shell. By further testing out this assumption, they reported that at 300 K, the shell does not affect the ferromagnetic behaviors of the core. Furthermore, they concluded that all the NPs showed almost identical temperature dependency at 300 K, indicated by the hysteresis loop under a low field [162].

### 3.3. Solvothermal Synthesis of MPNPs

Solvothermal synthesis is typically composed of a chemical reaction that takes place under high pressure and high temperatures (>180 °C) to generate a compound in a solvent. This ensures high-quality crystallinity of the products [163]. IONPs can be synthesized through this approach by reducing iron acetylacetonate (Fe(acac)_3_) with 1,2-hexadecanediol in the presence of oleic acid and oleylamine. This will produce small, stable, and monodisperse IONPs. Oleic acid and oleylamine play the roles of stabilizing agents, reducing agent (oleylamine), and promoters of gold deposition on the surface of the IONP [164]. Gold can then be deposited by reducing Au (CH_3_COO)_3_ in the presence of 1,2- hexadecanediol and a capping agent at temperature between 180 and 190 °C. However, temperatures have to be carefully manipulated to control the thermally activated partial desorption of the capping layer in the IONP, followed by the deposition of the Au shell on the IONP surface and then subsequently depositing the capping agent on the surface of the Au shell [165]. Zhang et al. used a solvothermal approach to synthesize Ag@Fe_3_O_4_ core/shell nanospheres by reducing AgNO_3_ and Fe (NO_3_)_3_ with ethylene glycol. Ag forms first due to its faster reduction rate, followed by Fe_3_O_4_ deposition, forming a core–shell structure. The shells with numerous small NPs are 60 nm-thick, whereas the uniform silver NP core is 70 nm [166].

Lim et al. synthesized MPNPs by using a two-stage approach for the formation of the core involving high temperatures decomposition. In the first stage, FeO(OH), oleic acid and 1-octadecene were heated (200 °C) and underwent reflux under flowing argon for 2 h. Afterwards, the temperature was increased to 320 °C and refluxed. This method produced low-saturation magnetization IONPs with a size distribution centered around 12 nm [167]. In the second stage, the IONPs were then coated with high-magnetization IONPs by mixing them with iron acetylacetonate, 1,2-hexadecanediol, oleic acid, and oleylamine to preserve narrow size distribution while achieving high magnetic response. The mixture had to be heated under argon flow at 100 °C for 30 min, followed by a temperature increase to 200 °C for 1 h, and then refluxed for 30 min at 300 °C, yielding IONPs of ≈18.2 nm. Afterwards, the IONPs were introduced to a mixture containing 11-mercaptoundecanoic acid (MUA), which serves as a binding bridge between the IO and the Au seeds (1.5 nm) thanks to its thiol group, which targets the Au surface, while the carboxylic acid group attaches to the IO surface. A gold plating was then formed by introducing hydrogen tetrachloroaurate, tetrakis (hydroxymethyl) phosphonium chloride, and potassium hydroxide solution. The solution was incubated overnight to form the IONPs decorated with Au seeds [167].

Although solvothermal synthesis offers precise control over size, morphology, and product purity, there are many potential drawbacks including complex setups, high-pressure reactors, solvents with high toxicity, long reaction times, and difficulties monitoring and controlling reaction processes in real-time, since the reactions tend to be under oxygen-free environments [168,169].

## 4. Surface Modification and Functionalization

In a core–shell structure, magnetic cores are generally coated with a material that provides the necessary chemical and physical properties to allow surface functionalization. The choice of the coating material largely depends on the intended application of the core–shell NP. Silica coating is widely used on MNPs due to its chemical stability, which prevents oxidation, reduces accumulation, and provides an inert surface [170,171,172,173]. Additionally, silica can easily be functionalized with functional groups like amines (-NH_2_) or thiols (-SH), serving as an anchor point for the attachment of metal shells like gold, silver, and copper [174,175,176]. Polymers like polyethylene glycol, polyvinyl alcohol, polystyrene, and polyethyleneimine can also be used as surface modifiers to help prevent aggregation and modify the surface to a hydrophobic or hydrophilic complex [177,178,179]. The coating layer is then exposed to metal precursors, leading to the formation of plasmonic shells through chemical interactions between the functional groups of the core coating agent and the shell.

Plasmonic nanomaterials, like AuNPs, have an efficient surface chemistry. As these materials tend to be stable, once the functional group becomes attached, they typically remain intact under various conditions and changes. As the shape and size of the substrate can be tuned, their surface area and reactivity can also be optimized, improving functionalization. When the surface area increases, more of the NP atoms will be exposed to the surface. Because more atoms are exposed at the surface, the reactivity increases, creating more active sites [180,181,182,183]. In some cases, such as drug delivery, packing large amounts of molecules will increase the functional group density, hence enabling the delivery of more drug molecules to the target site and possibly increasing therapeutic efficacy [184,185].

Metal particles like gold form strong bonds with thiol compounds through covalent interactions. The oxidative adsorption of the S-H bond at the gold surface results in the formation of an S-Au bond through the reduction of the hydrogen, allowing direct attachments to ligands and molecules [186,187,188]. It can also bind to -NH_2_ groups through interaction with the lone pair of the nitrogen atom [189,190,191]. However, this bond is weaker than the Au-S one [192]. As gold is highly resistant to oxidation and corrosion, it is used to create long-lasting and stable bonds for some of the functional groups. It is also highly biocompatible and non-toxic, which makes it a suitable candidate for surface modifications for biological applications where a long presence is required without interfering with other biological processes and causing adverse reactions. Other metals like silver and copper also form complex bonds with the same groups as gold. Out of the three metals mentioned, copper tends to be more prone to oxidation than gold and silver, which can ultimately affect the functional group’s stability and binding efficiency. Copper (Cu) readily oxidizes when exposed to air, resulting in the formation of copper oxide (CuO) or copper (I) oxide (Cu_2_O). This creates an oxidated surface layer that can hinder the formation of more stable compounds with other functional groups. Cu ions can also be toxic in some biological scenarios like cellular environment, causing oxidative stress by participating in Fenton-like reactions, thus producing ROS groups (hydrogen radicals HO•), which can damage cellular components like DNA, proteins, and lipids [193,194,195]. On the other hand, silver (Ag) is more reactive than gold and less prone to oxidation than copper, making it a material of choice for applications like catalysis, where high reactivity and low instability are desired. Although it is less susceptible to oxidation than Cu, it can tarnish when exposed to sulfur-containing compounds, which can affect surface functionalization [196,197]. For this reason, although it can form strong bonds with thiol groups, they are generally less stable than Au-S bonds. Ag surface modifications are also limited in some biological applications since Ag ions can become cytotoxic in high concentrations [198,199].

NPs must be biocompatible and stable in biological applications such as cancer diagnosis or therapy. PEGylation is a surface modification by polyethylene glycol (PEG) chains. In order to modify the NP surface, the PEG chain needs to have a reactive group, such as amines, carboxyl, and thiols, that can interact with the surface of the NPs. These functional groups will be covalently attached to the NP, forming strong bonds. The PEG chain can vary in length and density, which can affect the NP’s hydrodynamic size and surface charge. PEGylation reduces immunogenicity and toxicity in NPs. Additionally, the hydrophilic nature of the PEG chain reduces opsonization and clearance by the reticuloendothelial system, increasing the circulation time of the NPs in the bloodstream. Furthermore, it minimizes non-specific binding with some biological analytes, improving targeting efficiency and reducing aggregation [200,201,202]. Liu et al. presented a FePt@Fe_2_O_3_-PEG NP for efficient targeting of folate receptor-positive tumor cells. They also attached the anti-cancer drug DOX through hydrophobic absorption. The NPs prove to be an effective drug delivery vehicle while also serving as in vivo MRI contrast agents. Their group was able to create a low toxicity-dual platform capable of selectively targeting cancer cells while also accumulating in the tumor site to create more magnetic resonance contrast [203].

**Figure 5:**
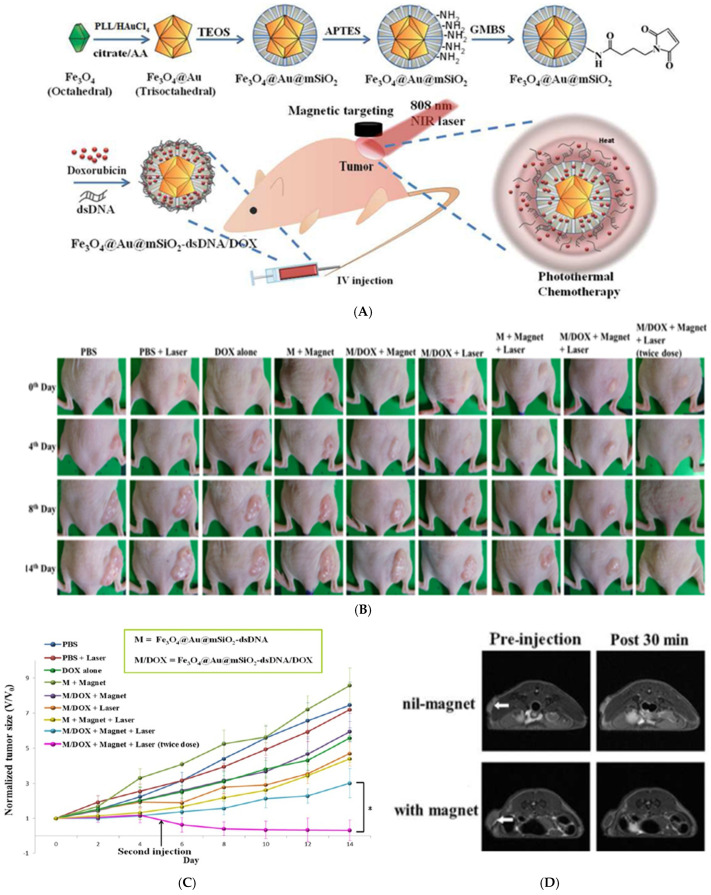
(**A**) Schematic overview of the experimental protocol for the multipurpose Fe_3_O_4_@Au@mSiO_2_-dsDNA/DOX NPs. (**B**) Images of mice population with different control groups and therapy approaches. NIR laser was applied for 30 min (808 nm diode laser at 3 W/cm^2^) and magnetic field was also applied for 30 min. (**C**) Tumor growth curve of the different sample groups. * *p* < 0.05 calculated and compared to once and twice dosed group. (**D**) T_2_-weighted MRI of the Fe_3_O_4_@Au@mSiO_2_-dsDNA/DOX NPs with and without exposure to external MF. White arrows indicate the location of tumor. Reprinted with permission from ref. [208]. Copyright (2014) American Chemical Society.

Oligonucleotides can be attached to plasmonic metals to develop biosensors for the detection of RNA/DNA sequences and proteins and as other bioanalytical tools for diagnosis and therapy [204,205]. These attachments are usually made using thiol, phosphorothioate, or amine modifications. Oligonucleotides can be modified at the 5′ or 3′ end with a thiol group creating molecules like 5′ or 3′-mercaptoalkyloligonucleotides, which can then covalently react with NPs, creating a stable functionalized surface [206,207]. Li et al. conjugated a polyhedral magnetic (Fe_3_O_4_)-trioctahedral Au shell that was further coated by a silica shell (mSiO_2_) with thiolated double-stranded DNA (dsDNA) and Doxorubicin (DOX) as a theragnostic platform for cancer (Figure 5A) [208]. They calculated the presence of approximately 12,830 dsDNA per core–shell NP. DOX anticancer drug was integrated into the Fe_3_O_4_/Au/mSiO_2_ NPs before the conjugation of the dsDNA to use the oligonucleotide to encapsulate the DOX. HeLa cells were inserted into the hypodermis layer of the thighs of nude mice. Once the tumor growth was complete, the mouse was treated with different spectra of therapies ranging from controls, drug-loaded nanoparticles, and a combination of drug-loaded NPs and NIR laser. Results showed that control samples had a tumor growth of seven times the initial size by day 14. In the case of the other sample populations, the tumor grew five to six times the initial size by day 14. Lastly, the sample that was treated once with drug-loaded NP + magnet attraction + laser saw a tumor growth of only three times the initial size by day 14. Another sample population was given a second dose of therapy by day 5, which resulted in a complete disappearance of the tumor by day 14 (Figure 5B,C). MIR experiments were conducted a week after the tumor growth. The NP platform was injected, and 30 min of magnetic attraction was applied after the injection. The results showed that the contrast signal of the tumor after the application of the magnetic field was reduced by 77.8%. On the other hand, no contrast changes were reported in the samples injected with the NPs, but no external MF was applied. This concluded that by applying an MF, the NPs accumulated at the tumor site, improving the negative contrast effects for MRI (Figure 5D).

Protein and antibody (Ab) functionalization is a key strategy for developing new diagnostic, therapeutic, and biosensors. Their binding strategies are also similar. The most common approach for Ab conjugation is to modify its primary amine with a thiol group through the use of a linker such as NHS-PEG-SH. The hydrolysis reaction releases the N-hydroxysuccinimide (NHS) in alkaline conditions (pH 7.2–9), creating a stable bond with the amine group. Afterward, a thiol tail is left readily available to interact with the substrate for surface modification [32,136].

Another way is by EDC/NHS chemistry. This approach is suitable for surface modifications, where the 1-ethyl-3-(3-dimethyl aminopropyl)carbodiimide (EDC) activates carboxylic groups at the surface of materials like silica. EDC is a zero-length crosslinker that reacts with carboxyl groups, forming unstable O-acylisourea intermediates. O-acylisourea has a short life span; thus, EDC is often combined with NHS. NHS stabilizes the reactive intermediates by forming NHS ester, creating more stable bonds while also reacting with primary amines [209,210]. In a study made by Talebzadeh et al., gold core–silica shell NPs of different shapes were functionalized with H_2_N-DNA by EDC/NHS chemistry. The conjugated core–shell NPs were used to monitor DNA hybridization through SERS probe detection [211].

Click chemistry introduces an azide-functionalized Ab to strain-promoted azide-alkyne cycloaddition (SPAAC), a reaction to functionalize alkyne-modified surfaces. This protocol is useful for biological applications where copper NPs are used, but the toxicity of the copper catalyst needs to be avoided [212,213]. Greene et al. synthesized a novel heterobifunctional linker that facilitated disulfide Ab conjugation to NPs. In order to attach the monoclonal antibody TRAZ to the NP, they opted for integrating a BCN-alkyne moiety due to its ability to interact with copper-free SPAAC. They reported a site-selective modification of the Ab Fab region by strained alkyne-pyridazinedione 3. Furthermore, by controlling the Fab direction, they reported stronger binding to human epidermal growth factor receptor 2 (HER2) compared to conventional NHS ester conjugation [214].

Physical absorption is another commonly used approach for Ab conjugation [215]. Ab can be absorbed onto the surface of some substrates through electrostatic, hydrogen bonding, hydrophobic interactions, or van der Waals forces. This approach does not require any chemical modifications of the Ab or substrate surface, but its attachments are weak compared to other protocols. Another disadvantage is the uncontrolled orientation of the absorbed Ab, which can lead to activity reduction. Nevertheless, some groups have studied the use of transition metal ions such as Zn^2+^ and Cu^2+^ as they promote binding to histidine-rich domains in the fragment crystallizable (Fc) region of Ab [216,217]. Zhang et al. led a study where they assembled NPs with site-specific antibodies for cell targeting. Ab, like Herceptin, was coated with zeolitic imidazole framework-8 (ZIF-8) in order to immobilize it on the NP surface and create a thin film coat. This allowed the control of the orientation of the Ab to selectively bind in the Fc region, allowing the Fab region to be exposed to target biomolecules. This experiment proved effective in controlling the Ab orientation and improving cell targeting. It also concluded that conjugations on Zn-based metal–organic frameworks (MOFs) increase by at least three-fold the available Fab regions compared to other MOFs like Co or Cu [218].

Biologically relevant molecules such as carbohydrates and peptides have emerged as the favorable functionalization agents to meet these properties. For example, maltose has been shown to be promising for enzyme immobilization [219]. L-asparaginase immobilized onto maltose functionalized Fe_3_O_4_@Au NPs lost only 10% of its activity in contrast to 50% for free L-asparaginase after 3 h incubation at 55 °C. The Km value of immobilized L-asparaginase decreased to 1.59 from 2.95 mM of free enzymes, indicating increased enzyme affinity for the substrate for the immobilized enzyme.

## 5. Applications of MPNPs for Cancer Diagnosis

### 5.1. Progress, and Challenges of MPNPs in Cancer Diagnosis

Over the past few decades, major progress has been achieved in cancer diagnosis, largely due to a combined effort from many researchers around the world. In the late 1990s and early 2000s, scientist pioneers demonstrated the potentials of gold nanoparticles as biological sensors, while SPIONs were introduced as contrast agents for MRI, enhancing tumor visualization [220,221,222,223,224,225,226,227,228,229,230,231,232,233,234]. In the same decade, NPs were also used as enhancers for PET, CT, and other optical imaging techniques. This led to increased sensitivity and specificity for early cancer detection. By the 2010s, nanoparticles were being used to develop personalized therapies through point-of-care devices, as well as for liquid biopsy applications such as CTC and exosome detection [235,236,237,238,239]. In the past few years, new emerging trends have given rise to new technologies such as the use of multimodal platforms like MPNPs, providing enhanced theragnostic applications, and the implementation of A.I in well-established technologies making diagnosis more precise. These advances have major impacts in the oncology field such as reducing the need for surgical tissue extraction though minimally invasive techniques, enhanced accuracy due to multifunctional NPs, and global accessibility thanks to the creation of portable nanotechnology-based diagnostic tools. Unfortunately, despite nanotechnology’s great potential, cancer diagnosis faces several challenges from technical, scientific, regulatory, and economic factors.

One of the biggest challenges that nanotechnology faces is the complexity of cancer biology. It is well known that cancer is highly heterogeneous in nature, with multiple stages and complex molecular profiles even among cancerous cells from the same origin. Furthermore, the tumor microenvironment can make targeting difficult due to differences in its pH, as well as immune response triggering. Additionally, some nanoparticle platforms can be difficult to consistently reproduce, which can affect performance and reliability in clinical settings. When it comes to clinical applications, many factors need to be taken into consideration, with one of the most important being safety concerns. Some nanoparticles such as those made of heavy or reactive metals can have adverse effects on patients’ health, mainly from metal poisoning or ROS reactions, which hinders biological and chemical processes. Lastly, the lack of standardized guidelines for evaluating nanoparticle-based diagnostics can also become a hurdle in clinical applications [240,241,242,243]. For these reasons, addressing some of these limitations can prove to be challenging but necessary. More research and long-term studies need to be conducted in order to learn more about the benefits of nanoparticles in the field of oncology. The Nanotechnology Characterization Laboratory (NCL) recently developed a standardized protocol for nanoparticle physicochemical characterization as well as preclinical testing of the immunology, pharmacology and toxicology properties. The idea is to aid researchers and developers in transitioning their concepts from the discovery to the clinical trials [244].

When it comes to MPNPs in cancer diagnosis, conceptualization came about in the 2000s and the proof of concept sometime between 2005 and 2010, with some of the earlier application as a multimodal agent around the same time [245,246,247,248]. Recently, their use has been explored for personalized medicine and early cancer detection in all-in-one devices capable of enriching biomarkers, magnetically capturing them, and optically characterizing them [136]. The latest introduction of A.I and machine learning has boosted image analysis and data interpretation accuracy [138].

The usage of MPNPs is still limited due to the presence of some challenges such as complex synthesis and reproducibility. Some groups have addressed these problems by incorporating machine learning while automating the synthesis process, yielding highly uniform NPs while increasing reproducibility [249,250,251]. As we continue to expand on some of the topics of interest in this review article, we will go into depth exploring the previously mentioned challenges, applications and usefulness of MPNPs in cancer diagnosis.

### 5.2. Magnetic Resonance Imaging (MRI)

MRI techniques are dependent on the proton density of spin–lattice relaxation (T_1_) and spin–spin (T_2_) relaxation time. The magnetic core of MPNPs can enhance the relaxation time of T_1_ and T_2_. In MRI, a T_1_-weighted image is one of the most basic pulse sequences for producing a variety of T_1_ relaxation times in the analyzed tissue. It relies on the longitudinal relaxation of the tissue’s net magnetization vector. This makes the longitudinal spins on the static magnetic field (B_0_) change to a transverse plane through radiofrequency (RF) pulses. This causes the protons in the water molecules within the tissue to become excited. The relaxation of the protons happens when the RF is turned off and before it is applied again. This period is known as the recovery phase, causing the protons to gradually realign with the MF. Through this method, tissues with short T_1_ relaxation time, like Fat, recover their longitudinal magnetization faster, thus appearing brighter, whereas body fluids like cerebrospinal fluid (CSF) or edematous tissue exhibit longer relaxation times and appear darker [252,253]. Alternatively, T_2_ occurs when energy is exchanged between the magnetic dipoles and the analyzed tissue’s local magnetic field. In other words, it is the time it takes the protons to lose their phase coherence in the transverse plane, resulting in a signal intensity loss. T_2_ occurs immediately after the RF pulse is turned off, and during that time, the protons begin to lose their coherence in the transverse plane. In applications like T_2_-weighted imaging, the timing of the echo (TE) that appears between RF pulses and MRI signal measurements is chosen to visualize different T_2_ relaxation times between tissues. Tissues expressing longer T_2_ times, like CSF and edematous tissue, appear brighter, while tissues with lower T_2_, like muscles and fat, appear darker [254,255]. MNPs can help enhance the relaxation time of both modes, creating darker or brighter MRI contrast. The magnetic core dynamically interacts with the hydrogen nucleus of the water molecules in its surroundings by producing induced magnetization, which affects the distribution and uniformity of the magnetic field. This, in contrast, affects the relaxation time of the protons around them [256].

**Figure 6:**
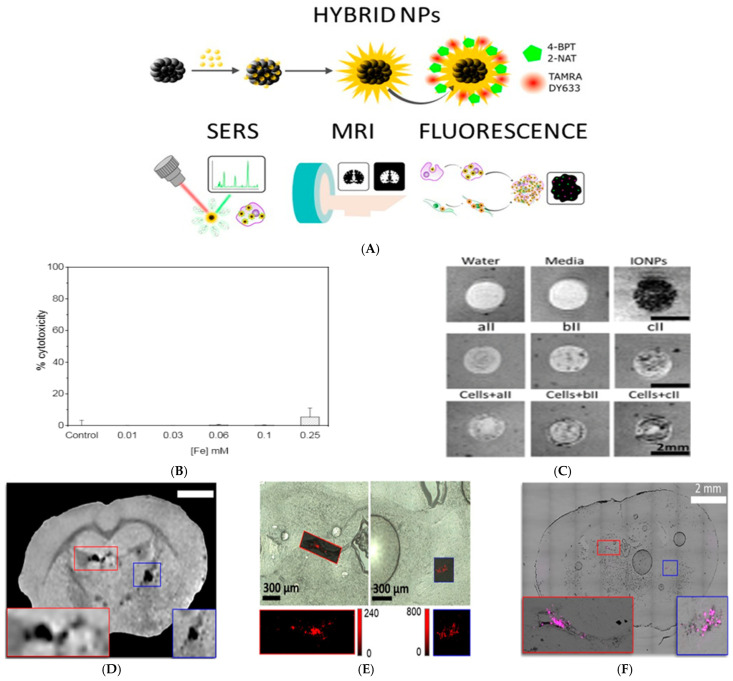
(**A**) Overview of the usage of the iron oxide core–gold shell nanostars functionalized with Raman nanotags 4-BPT or 2-NAT, and FL dyes TAMRA or DY633 for multimodal (MRI, SERS, and FL) imaging. (**B**) MPNPs biocompatibility test of MCF-7 BC cell line by LDH showing low cytotoxicity after 48 h of NP exposure, even at high metal concentrations (0.25 mM). (**C**) MPNPs tested as a contrast agent for MRI under different conditions. Serie II (aII, bII, and cII) are composed of small MPNPs that maintain well-defined spikes. (**D**) MRI image of the spiked mouse brain for ex vivo modeling showing dark spots where the MCF7-MPNPs and the control MPNPs were injected (right hemisphere = blue boxes in both panels (**D**,**F**). (**E**) SERS and (**F**) FL imaging of the brain tissue. Reprinted with permission from ref. [257]. Copyright (2022) American Chemical Society.

Plasmonic materials are known for their optical properties, and although they do not exhibit the same magnetic properties as MNPs, they have found their way into MRI applications. The integration of plasmonic materials is typically seen in the form of hybrid NPs, combining a magnetic core with a plasmonic shell. This allows a multimodal application for optical and magnetic resonance imaging with the potential of being further used for therapy. The plasmonic shell can be used to enhance contrast in optical imaging techniques such as photoacoustic imaging or SERS, while the magnetic core enhances the contrast in MRI. It can also help improve biocompatibility, reduce toxicity, and increase stability in MRI without sacrificing the magnetic enhancement. De la Encarnacion and colleagues developed a colloidal system of hybrid magnetic–plasmonic NPs that act as a contrast agent for multimodal SERS, MRI, and fluorescence imaging (Figure 6A) [257]. The MPNP surface was functionalized with Raman molecules 4-biphenylthiol (4-BPT) and 2-naphthalenethiol (2-NAT) for SERS encoding and TAMRA and DY633 fluorophores for fluorescence imaging. To measure the cytotoxicity of the NPs, different concentrations ranging from 0 to 0.25 mM were spiked into BC cell line MCF-7. After 48 h, cytotoxicity analysis by lactate dehydrogenase (LDH) assay and membrane permeability staining was conducted on the cells. No cytotoxicity was observed in the concentration ranges of 0.01–0.1 mM, but at 0.25 mM, the cell viability reached <10% (Figure 6B). They concluded that the NPs were highly biocompatible, making them suitable for biomedical applications. MRI T2 mode was then conducted on the cells to test the contrast agent properties of the NPs. As seen in Figure 6C, only the samples containing the MPNPs or IONPs appeared with a dark contrast. Ex vivo experiments were conducted through intracerebral injection into an excised mouse brain to test the NPs as contrast agents in multimodal imaging. The experiment first started by incubating MCF-7 cells with the MPNPs@2-NAT@DY633 at a final total concentration of 0.05 mM, which was previously used for MRI imaging. The cells were then trypsinized and concentrated at 2 × 10^6^ cells/mL, followed by injecting 5 µL into the brain’s right hemisphere. MPNPs@2-NAT@DY633 were then injected into the left hemisphere as a control. Although the whole brain was used in MRI imaging, it was later sliced to collect the tissue for FL and SERS imaging. Results showed that dark spots could be seen by MRI where both the MPNPs control and MPNP-endocytosed MCF-7 were injected (Figure 6D). Moreover, SERS (Figure 6E) and FL images (Figure 6F) were able to detect the NPs and create a map of the brain tissue affected by the cancer cells. They concluded that the NPs were suitable for multimodal applications in ex vivo models by acting as a contrast agent for three different imaging techniques.

### 5.3. Photoacoustic (PA) Imaging

PA imaging is a noninvasive technique that combines high-contrast optical imaging with high ultrasound spatial resolution. A laser sends short pulses into the tissue at a wavelength that matches the LSPR of the plasmonic NPs. The NPs absorb the light and undergo rapid thermal expansion due to the conversion of the energy from the photons into heat. The thermal expansion then generates ultrasonic waves that propagate through the tissue and be detected by a transducer to produce an image. Thus, the PA image provides information about the optical absorption properties of the tissue, which can reveal structures that otherwise might be invisible with other traditional techniques. Due to the rapid generation of ultrasonic waves upon the absorption of the laser by the NP, the PA imaging can further be performed in real-time, allowing the monitoring of dynamic processes such as blood flow or drug delivery [258,259].

When the MPNPs interact with the laser beam at the resonating wavelength, the particles absorb the light more efficiently than the surrounding tissue as with the plasmonic NPs and thus enhance the photoacoustic signal. As mentioned earlier, the metallic shell can be engineered by changing the thickness, shape, or composition used to tune its LSPR for maximal laser absorption. By tuning this physical property, the LSPR can be generated in the NIR region where biological tissue has minimal absorption. Thus, the MPNPs can be used for deep tissue PA imaging. An example of PA imaging with MPNPs was demonstrated by Huang et al. using γFe_2_O_3_@Au nanoflower (MG-NFs) [260]. In this study, the MG-NFs were synthesized through iterative growth of the Au shell on the γFe_2_O_3_ NPs. PA effects were tested with an 808 nm laser, which showed high PA contrast, offering the potential for application in clinical settings. In addition, they determined that the T_2_-weight relaxivity of the MG-NFs was much higher than that of γFe_2_O_3_ NPs (76.25 ×10^−3^ M^−1^ s^−1^ for MG-NFs versus 46.53 × 10^−3^ M^−1^ s^−1^ for γFe_2_O_3_ NPs). The MG-NFs also showed greater SERS enhancement of the 4-MT Raman that solid AuNPs. The NFs proved to be a valuable tool as a theragnostic agent thanks to its ultrasensitive SERS, high-resolution PA, and real-time MRI.

### 5.4. Surface-Enhanced Raman Scattering (SERS) Detection

Another major diagnostic application of MPNPs is SERS detection, similar to plasmonic NPs. SERS has emerged as a powerful tool in cancer diagnosis, offering high sensitivity and specificity for detecting cancer biomarkers in various biological samples. The principle of SERS with MPNPs relies on the electromagnetic and chemical enhancements by the plasmonic shell that can be further enhanced with anisotropic shells. Recently, we developed a multiplexed SERS method for CTC surface marker detection using magnetic multicolor SERS nanotags with IO-Au core–shell nanopopcorns (NPCs) in conjunction with a microfluidics-based immunomagnetic separation [136]. Four Raman nanotags with distinctive fingerprint spectra were separately coated to the NPCs and conjugated with antibodies to report four different surface protein markers on cancer cells. A microfluidic device was designed to capture the NPC-bound cancer cells on a chip magnetically and then fluorescently stain cell nucleus and white blood cells (WBCs). The level of each targeted protein was obtained by signal deconvolution of the mixed SERS signals from individual cancer cells. EpCAM, HER2, CD44, and IGF1R were simultaneously detected and distinguished on four three different cancer cells lines (SK-BR-3, MDA-MB-231, and MCF-7). This method is strongly correlated with ELISA, with high correlation coefficients (r ≥ 0.97) and low p-values (*p* < 10^−6^). This microfluidics-based multicolor multiplexed SERS method combined with immunomagnetic separation has the potential to more sensitively detect and monitor CTCs for metastasis detection and monitoring.

**Figure 7:**
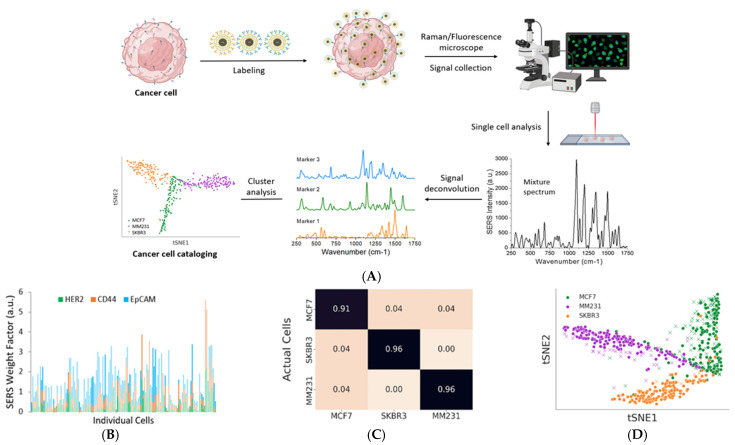

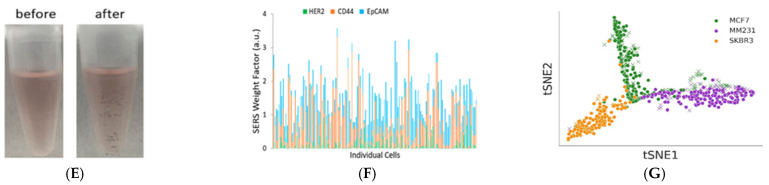
(**A**) Schematic of multiplexed surface protein detection and classification of cancer cells with IO/Au/GERTs. (**B**) SERS weight factors for each protein marker from 150 SKBR3, MM231, and MCF7 mixed cells. (**C**) Confusion matrix from the training data with the actual cells along the *y*-axis and the predicted cells along the *x*-axis. Values are presented as fractions. (**D**) tSNE visualization of the cancer cell training data along with the prediction data for mixed SKBR3, MM231, and MCF7 cells. (**E**) Magnetic separation of mixed cancer cells (MM231, SKBR3, and MCF7) from blood after labeling with HER2, EpCAM, and CD44 conjugated IO/Au/GERTs. (**F**) SERS weight factors for each protein marker from 150 SKBR3, MM231, and MCF7 mixed cells separated from whole blood. (**G**) tSNE visualization of the cancer cell training data along with the prediction data for mixed SKBR3, MM231, and MCF7 cells isolated from blood. In (**D**,**G**), crosses represent predicted data and dots represent training data. Training and prediction data use the same colors for the same cells. Reprint with permission from ref. [138]. Copyright © 2024 American Chemical Society.

AI has emerged as a changing force in modern medicine, improving the accuracy and efficiency of cancer detection and diagnosis. The integration of MPNPs with machine learning techniques offers a promising approach in cancer detection. This innovative methodology boosts the unique properties of MPNPs, which, together with AI, enhance the sensitivity and specificity of cancer diagnostics. The application of these nanoparticles in biomarker detection is increasing in recent research, highlighting their potential to revolutionize cancer detection techniques. Recently, we developed a cancer diagnostic approach by classifying three subtypes of BC cell lines using machine learning based on their surface protein expression (Figure 7) [138]. Gap-enhanced IO-Au core–shell NPCs with a Raman tag embedded between the inner and external layer of Au were prepared and linked with target specific antibodies. Three Raman tags with distinctive Raman spectra were used to report three different protein markers on individual cancer cells, with QSY21 for CD44, BHQ3 for EpCAM, and QXL680 for HER2 (Figure 7A). The expression level of each protein on each cell was determined by signal deconvolution with CLS of the mixture SERS spectrum from the induvial cells. These data were used as the training data to build a random forest model to classify different cancer cell lines in the sample containing mixed cancer cells. The accuracy of this method in multiplexed detection and cell classification was validated with mixed BC cancer cells of MDAMB231, SKBR3, or MCF7 cells suspended in PBS (Figure 7B–D) and in blood (Figure 7E–G). The random forest model produced an average F1 score of 0.94. The model correctly predicted 91% of MCF7 cells, 96% of SKBR3 cells and 96% of MM231 cells. This study shows that AI can help predict unknown cancer cells and potentially identify the origin of CTCs in the blood system.

### 5.5. Multimodal Imaging

Advancements in multimodal imaging have revolutionized the medical diagnostic field by enabling the integration of different techniques to provide more information and better assessment in diagnosing cancer. Multimodal imaging-based diagnosis has made its way into hospital settings. Dr. Ferenc Jolesz and Clare Tempany at the Brigham and Women’s Hospital (Boston, MA, USA) designed an advanced multimodal image-guided operating (AMIGO) suite composed of an MRI, operation room (OR), PET, and CT interconnected sterile rooms. The goal of this research was to test clinical translational research ideas through rigorous, transparent, and reproducible experiments. This was achieved through the incorporation of multiple imaging modalities to assess the combination of pre-procedures and intraprocedural imaging during surgical intervention, enabling the acquisition of accurate information on the tumor, thus ensuring in situ destruction, complete disposal of the affected tissue, or target-specific biopsy [261]. A group led by Brennan et al. showed that Fe_3_O_4_-Au core–shell NPs with 4 nm shell gave high contrast in dual dark-field imaging and coherent anti-Stokes Raman (CARS) microscopic imaging of cancer cells [262]. It was also demonstrated that at the concentration used (100 mg/mL), there were no signs of cytotoxicity in the cells, proving that the NPs also had high biocompatibility.

### 5.6. Biosensing

The development of biosensors has revolutionized cancer diagnosis as a highly sensitive, real-time, and noninvasive approach for the detection and monitoring of cancer biomarkers. They prove very useful in early detection, personalized treatment, and disease monitoring, thereby helping in strategic management to obtain better outcomes for cancer patients. Because they can be miniaturized for point-of-care testing and liquid biopsy applications, they can be ideal for usage in scenarios where more complex diagnostic tools are difficult to access. MPNPs are used as sensing probes thanks to their ease of surface functionalization, biocompatibility, and the lack of magnetic susceptibility from biological markers. Many of the biosensor platforms using nanomaterials are based on optical detection. Optical techniques mainly include colorimetric, luminescence, fluorescence, SPR changes, and Raman approaches. These techniques depend on changes in the intensity, phase, wavelength shift, and angle of reflection [263,264]. Fluorescence (FL) is one of the most commonly used methodologies relying on the labeling of a targeting molecule with a fluorescent contrast agent such as dye, fluorescent protein, and QDs. An example is the sensing of CTCs in whole blood with star-shaped MPNPs by Fan et al. [265]. In this study, Fe-Au NPs were modified with Cy3-S6 aptamer to detect HER2 on SK-BR-3 breast cancer cells. Combing magnetic separation, the nanoprobe can detect the cancer cells even in 0.001% mixtures. Combing PTT, this nanoplatform shows enormous potential as a theragnostic agent for cancer detection and treatment.

Optical sensors for nucleic acid or protein markers typically use AuNPs with a targeting ligand to pull down the targets to a surface biosensor and then digitally count each the captured NPs. However, this type of assay needs a long response time due to the transportation of the analytes or nanotags to biosensor surface by diffusion. Che and colleagues developed a magnetic activate capture and digital counting (mAC + DC) approach to accelerate single molecule sensing using a photonic crystal (PC) sensor and spiky Fe_3_O_4_-Au core–shell NPs [266]. Fe_3_O_4_-Au NPs with target-specific probes bound to miRNA target and were driven to the PC surface within one minute by magnetically driven transport. By matching the LSPR of the Fe_3_O_4_-Au NPs with the PC resonance, the captured MPNPs were individually visualized via resonance reflection. The method showed a limit of detection (LOD) of 61.9 aM for miR-375 cancer marker in unprocessed human serum samples, with a broad dynamic range (100 aM–10 pM).

## 6. Applications of MPNPs for Cancer Treatment

### 6.1. Photothermal Therapy (PTT)

One of the main concerns in cancer patients is the dissemination and spreading of cancer to other healthy tissues and organs through metastasis. The metastasis cascade starts with the detachment of cancerous cells from the primary tumor and the extravasation into the circulatory system and surrounding tissue. Once in the bloodstream, the cancerous cells can camouflage from the immune system through different pathways. These circulating tumor cells will then anchor into distant tissue and create secondary tumors. The cycle will keep repeating toward other distant organs and tissues until the patient succumbs to the disease. Metastasis is the cause of 90% of deaths by cancer [267,268].

PTT has gained attention in recent decades thanks to its minimally invasive and localized attributes. PTT relies on the conversion of light energy, typically deep-penetrating NIR light, into heat to destroy cancerous tissue. At 41 °C, cancer cells start experiencing thermal stress and activate their heat shock proteins (HSPs) in order to protect themselves. However, prolonged exposure overwhelms the protective mechanism, causing cellular death by affecting their protein folding abilities and enzymatic activities. Other effects that take place are hyperthermia, metabolic dysfunction, impaired DNA reparation, disruption of the tumor microenvironment, and immune response [269,270]. Nonetheless, to make PTT work effectively, a temperature of 50 °C or higher at the tumor site needs to be reached. The center of the tumor often exhibits a hypoxic microenvironment due to inadequate blood supply. This can make the tumor resistant to other therapy approaches such as chemo- and radiotherapy. However, the extreme internal temperature by PTT can induce irreversible and immediate cellular damage, leading to necrosis. Additionally, the proteins inside the tumor will suffer from thermal coagulation through protein denature. Reaching high temperatures overwhelms the tumor’s natural defensive mechanism, thus ensuring complete ablation and possibly reducing recurrence risks [271].

Similar to plasmonic NPs, MPNPs can be used for PTT owing to their photothermal property. For example, Ghaznavi et al. developed IO-Au core–shell NPs for targeted PTT with folic acid (FA-PEG-Au@IONP) [272]. The IO NPs had a diameter of 50 nm with a 10 nm Au shell. In the in vitro studies, nasopharyngeal carcinoma KB cells and breast adenocarcinoma MCF-7 cells were incubated with the nanocomplex (50 ug/mL, 12 h) and laser irradiation (808 nm, 6 W/cm^2^, 10 min). Significant cell death was observed for both KB (∼62%) and MCF-7 (∼33%) cells following PTT. It was also found that the majority of the cell death was due to apoptosis.

PTT can also be combined with other treatments to improve efficacy. For example, Hu et al. reported the use of Fe_3_O_4_@Au core–shell NPs for combined radio-photothermal therapy to treat cervical cancer [273]. Their studies showed that the HeLA cells incubated with Fe_3_O_4_@Au NPs (12.5 ug/mL, 12 h) showed a survival rate of 40.2% with X-ray irradiation alone. Combing PTT with NIR radiation, the survival rate decreased to 12.7%. Bhana et al. developed IO-Au NPCs for magnetically amplified PTT and photodynamic therapy (PDT) [274]. In this study, the therapeutic efficacy of cancer cells had a dual amplification: magnetic field enhanced drug delivery and combinational PTT/PDT. As demonstrated in the work with KB-3-1 cells as an example, the PTT and PDT (silicon 2,3-naphthalocyannie dihydroxide as the photosensitizer) alone gave cell survival of 49.1% and 70.9%, respectively. However, the PTT/PDT combination treatment resulted in cell survival of 26.3%. With magnetic-field-assisted drug delivery, the cell survival rate decreased to 7.1%. This “three-in-one” nanocomplex shows strong potential to delivery therapeutic agents deep into a tumor through magnetic attraction and completely eradicate tumors with PTT and PDT.

PPT has also been combined with imaging modalities for theragnostic application using MPNPs. As mentioned above, Fan et al. linked Cy3 to star-shaped Fe-Au NPs for fluorescence imaging and PPT of CTCs in combination of CTC isolation with magnetic separation [265]. Abded developed a multifunctional theragnostic approach for colorectal cancer using IO-Au core–shell NPs [275]. MRI with T_2_-contrast was used to image the tumor accumulation of the IO-Au NPs after intertumoral (I.T) or intravenous (I.V) administration into Balb/c mice bearing CT26 cancer cells. With I.T. NP administration and magnetic drug targeting (MDT), a temperature rise of 16.7 °C was achieved with laser irradiation to induce PTT-inhibited tumor growth up to 66%. On the other hand, the tumors that were treated with I.T injection + NIR radiation and I.V injection + MDT + NIR radiation were completely eliminated within a few days after the treatment started, allowing the mice to remain healthy for nearly a month of follow-up periods and showing no signs of recurrence.

### 6.2. Magnetic Hyperthermia (MH)

Similar to PTT, MH uses heat to destroy tumor cells. The amount of heat generated by the NP is proportional to the area of the hysteresis loop during one cycle of MF. When the MNPs are exposed to an alternating magnetic field (AMF), energy is dissipated through the hysteresis loss, which is correlated with how quickly the magnetization of the NPs’ single or multi-domains follows the AMF changes. Also, when an external MF is applied, the internal magnetism vector of the MNPs changes to align with the direction of the external field. In AMF, the direction of the magnetic field vector $\vec{H}$(t) is changing periodically in both magnitude and direction. This correspondingly causes the magnetic moment ($\vec{m}$) of the MNP vector to change. Superparamagnetic NPs can rotate their magnetic moments without physically rotating the NPs, which is known as Néel relaxation. In addition to Néel relaxation, MNPs can also exhibit Brownian relaxation. In contrast, this type of relaxation depends on the external rotation of particles to align its magnetic moment with the external field. On the other hand, ferromagnetic NPs suffer from hysteresis loss due to having larger and more stable magnetic domains, which does not allow the magnetic moment to immediately follow any external MF direction. As the $\vec{m}$ lags behind the external field, energy is lost during each of the AMF cycles that contribute to heat generation.

Nevertheless, MNPs can generate heat through Néel and Brownian relaxation to kill cancer cells through hyperthermia [276,277]. Gharibkandi et al. reported the use of superparamagnetic IO NPs coated with radioactive ^198^Au (SPION@Au) for dual magnetic hyperthermia and radionuclide therapy [278]. The SPION@Au core–shell NPs exhibited a magnetization saturation of 50 emu/g. This allowed the core–shell NPs to reach a temperature of 43 °C at an MF frequency of 386 kHz. Using HepG2 cell line as the hepatocellular carcinoma (HCC) model, they determined that MH with β^−^ radiation by ^198^Au induced high cell cytotoxicity, with a cell survival fraction below 8% for 2.5 MBq/mL of radioactivity after 72 h of treatment. Another study by Tonthat et al. involves the use of Fe_3_O_4_@Au NPs as a theragnostic agent for MH and CT imaging [279]. The Fe_3_O_4_@Au NPs reached a CT value of 851 HU, much higher than those of Fe_3_O_4_ (158 HU). As a therapeutic tool, the Fe_3_O_4_@Au NPs reached a heat efficiency of 43–45 °C, sufficient for tumor hyperthermia therapy.

### 6.3. Drug Delivery

Many currently available materials for drug delivery face several challenges, including poor biocompatibility and in vivo stability as well as lack of target specificity [280]. Additionally, the common cancer therapies (chemotherapy and radiotherapy) produce severe side effects, especially inducing the damage of healthy cells. Many nanoplatforms have been developed to address these challenges by making the use of the intriguing physical and chemical properties of the nanomaterials. NPs can deliver the drugs preferentially to tumor via EPR effect while reducing toxicity and drug degradation of free drugs to improve therapeutical efficacy [281]. MPNPs have proved to be a great candidate as a drug carrier because of their ease of conjugation, great biocompatibility, and low cytotoxicity. They can be used for combination treatment. For example, Darsajini et al. synthesized Fe_2_O_3_ @Au/alginate hydrogel as a carrier for cisplatin for combined chemotherapy and PTT [282]. The Fe_2_O_3_ @Au/alginate hydrogel loaded with the cisplatin at a concentration of 40 μg/mL reported a significant loss of KB cell viability by almost 90% after being irradiated for 5 min. To further enhance treatment, the magnetic core allows for the use of an external MF to guide the NPs to the cancer cells or tumor site [274].

**Figure 8:**
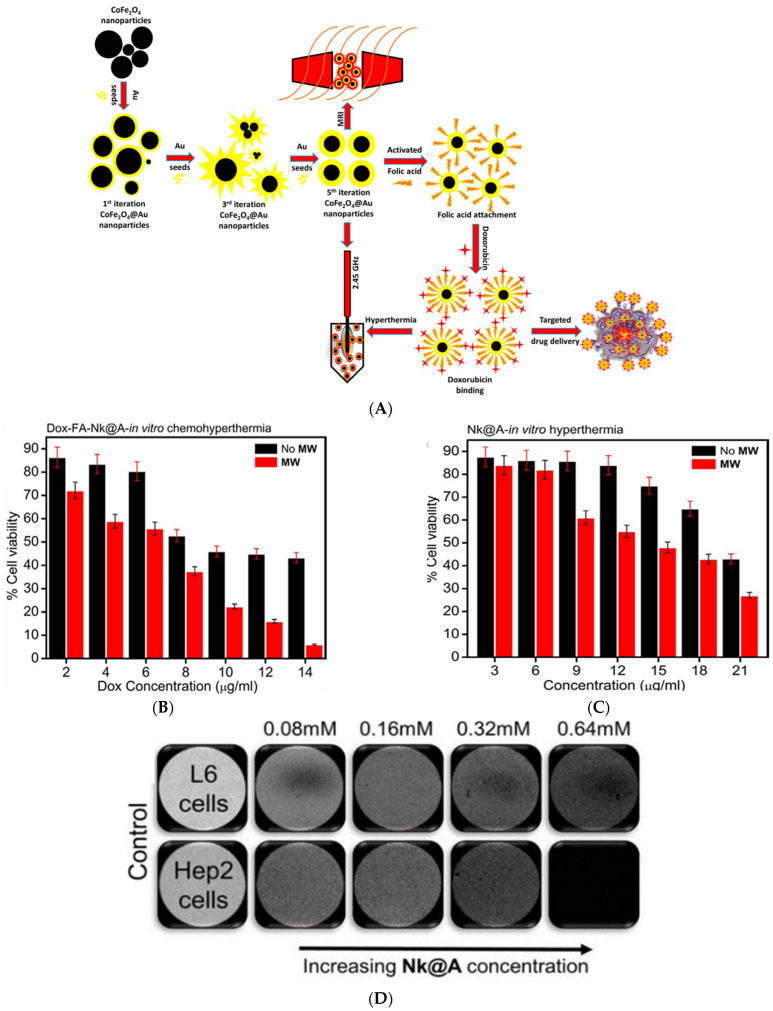
MPNPs for MRI, hyperthermia, and drug delivery. (**A**) Schematic of MRI and chemohyperthermia therapy using Dox-FA-Nk@A-MW. (**B**) In vitro chemohyperthermia therapy showing cell toxicity at different concentrations of the Dox-FA-Nk@A in compared to the treatments without MW. (**C**) In vitro hyperthermia therapy showing cell cytotoxicity at different concentrations of the Nk@A in compared to the treatment without MW. (**D**) MRI T_2_-weighted image of L6 cells and Hep2 cells using Nk@A at different concentrations. Reprint from ref. [283]. Copyright 2016 Springer Nature Limited.

Most often, MPNPs are used as a multifunctional platform for cancer detection and treatment. Ravichandran et al. synthesized a Au-seeded coated CoFe_2_O_4_ nanokernel (Nk) to deliver doxorubicin (Dox) with folic acid (FA) as the targeting agent to cancer cells for hyperthermal therapy, chemotherapy and MRI (Figure 8A) [283]. By heating the therapeutical agent (A) with microwaves (MW) (2.45 GHz, 50 s), the chemo-hyperthermal effect increased the interaction between the anti-cancer drugs and the cancerous cells, hence enhancing cellular drug uptake, DNA damage, and decreasing DNA reparation capabilities. The thermal enhancement from the Dox-FA-Nk@A-MW resulted in a half-maximal inhibitor concentration (IC50) of 8 ug/mL (Figure 8B), whereas Dox-FA-Nk@A-MW resulted in an IC50 of 12 ug/mL (Figure 8C). This concluded that the thermal enhancement provided by the Nk towards Dox effectively inhibited cellular viability at increased temperatures and low drug concentrations while also serving for MRI by providing high relaxation times as a T_2_ agent in the presence of L6 and Hep2 cells (Figure 8D).

### 6.4. Multimodal Therapies (MH and PTT)

Due to the physicochemical properties of MPNPs, some groups have explored their potential as a dual therapy platform for cancer therapy [284,285,286]. Researchers have reported using MPNPs for a combination of PTT and MH. Espinosa et al. conducted in vivo experiments and reported that the NPs were subjected to both magnetic (900 kHz and 25 mT) and laser (680 nm at 0.3 W/cm^2^) stimulation [287]. When treated with MH only, the temperature on the tumor site increased by 6 °C in all samples. During laser exposure, the temperature increase in each sample ranged from 2 to 6 to 11 °C. When both approaches were combined, the temperature for sample 1 increased 14 °C, while sample 2 and 3 increased up to 18–19 °C. Furthermore, the thermal efficiency in the combined mode increased as the concentration increased, reaching 17 °C in 100 s. On the subcutaneous tumor site, 50 µL of the MPNPs were injected, while on the collateral tumor, no MPNPs were used as a form of negative control. When the tumor was treated with individual approaches, the thermal performance showed temperature increases of 9–10 °C, while no temperature increase was observed in the control tumor. Under the combined therapy, the temperature increased by almost 20 °C within 2 min of exposure. Additionally, after 3 days, the heating performance (heat maintained) was still 80%, proving its lasting effects. Finally, during the 5-day follow-up, tumor regression on the treated site was revealed, in contrast with the control [287].

Espinosa et al. further explored the capabilities of MPNPs in another experiment. Two NPs, one with a magnetic core size of 16 nm and another of 20 nm, were subjected to alternating MF at 470 kHz and 18 mT to assess their MH capabilities [288]. Afterwards, the NPs were irradiated with a 680 nm laser at 0.5 W/cm^2^ to observe their PTT properties. Lastly, a combination of MH and PTT was conducted as well. For the 20 nm core NP, the temperatures at 24 mM [Fe] concentration increased by ≈5 °C, while for 16 nm, at the same concentration, it increased by ≈10 °C. For PTT, the 20 nm NP reached a maximum temperature of ≈28 °C at [Fe] 12 mM, while at 24 mM, no difference in temperature was observed. As for the 16 nm, at 12 mM, the ∆T increased by ≈23 °C while reaching the highest ∆T of ≈27 °C at a concentration of 48 mM. For the dual performance, the 20 nm NP achieved a ∆T increase of ≈34 °C at [Fe] 24 mM, while the 16 nm one at the same concentration reached approximately the same temperature. The NPs were the then incubated with CT-26 cells to observe their thermal properties and their effects in vitro. The cells labeled with the 16 nm core NP showed that for the MH the cell viability was still ≈100% after leaving them for 24 h after treatments (exposure of 15 min). As for PTT, the cell viability dropped to 20%. Nonetheless, for the dual approach, the cell viability reached a staggering 3–4%. After in vitro experiments, they tested the NPs in vivo by magnetically guiding the NPs towards the tumor site and applying dual therapy. In their experiments, a sample was guided using external MF, while another sample was not guided magnetically, and one more was non-injected butt-irradiated with a laser, and finally, one more acted as the control. For the guided sample, the ∆T on the tumor site reached 20 °C, while that for the non-guided one was ≈13 °C, and the laser-only sample was ≈10 °C. This indicated an accumulation of the NP on the tumor site by guiding them with an external MF. After a 7-day follow-up, the control tumor size increased to almost four times its original size, while the laser-only one increased almost three times, the non-magnetic guided one increased by almost two times, and finally, the magnetic-guided sample saw a tumor size decrease [288].

### 6.5. Numerical Work on Thermal Therapies

The utilization of MPNPs in PTT and MH has gained momentum in the past few years. As these NPs are composed of a magnetic core and a plasmonic shell, they can exhibit enhanced heating capabilities when subjected to a combination of alternating MF and laser irradiation. The fundamental mechanism behind their heating efficiency can be explained by Néel and Brownian relaxation. When an alternating MF is applied, the NPs can produce energy which is then converted into heat; nonetheless, with time, the NPs can lose heat due to the relaxation loss of the magnetic moment. In the Néel relaxation, the NP does not move, but rather the rotation or direction of the magnetic moments inside the NP changes. On the other hand, in Brownian relaxation, the NP structure rotates against resistance from its surrounding medium [289]. These relaxation times can be modeled as seen in Equation (5):

(5) $$\tau_{B}=\frac{3_{\eta_{0}}V_{h}}{k_{B}T},\tau_{N}=\frac{1}{f_{0}}\exp\left(\frac{K_{a}V_{c}}{k_{B}T}\right),\;$$

where $\tau_{B}$ is the Brownian relaxation time, $\tau_{N}$ the Néel relaxation time, $\eta_{0}$ represents the viscosity of the medium, $V_{h}$ is the hydrodynamic volume of the NP, $V_{c}$ is the volume of the magnetic core, $K_{a}$ represents the anisotropic constant, $k_{B}$ is the Boltzmann constant, T is the absolute temperature, and $f_{0}$ is the attempted frequency for changes in the dipole moment (≈10^−9^). Unfortunately, this equation is only applicable for small magnetic fields and negligible particle-particle interactions. Furthermore, the effective relaxation mechanism and time are given by $\tau_{eff}^{-1}\equiv \tau_{B}^{-1}+\tau_{N}^{-1}$, thus showing the dependence of the Brownian and Néel relaxations on the particle diameter, medium viscosity, and anisotropic constant values [290].

Various groups have conducted numerical studies on plasmonic and magnetic oxide materials for simulated application in thermal cancer therapies, although almost no literature could be found on magneto-plasmonic core–shell NPs numerical studies. When it comes to plasmonic materials, Kim et al. ran various simulations on heat transfer of different shapes of gold nanoparticles for PTT and their potential in the death of squamous cell carcinoma [291]. DDA was used to calculate the optical efficiency of the AuNPs, while the Pennes bioheat equation was used for thermal analysis on biological tissue. This equation considers that the heat generated by the blood and metabolism is being uniformly generated in the biological tissue. Following validation of the simulation, they proceeded to perform a numerical analysis of PTT on a skin structure composed of four layers which included the carcinoma, followed by the injection of six AuNPs of different shapes (rod, sphere, shell, pyramid, prism, and cube). The results showed that when the volume fraction of the AuNR in the tumor was high ($f_{v}$ = 10^−3^), the coefficient of the absorbed light of the tumor tissue and the NP was also high. This indicates that a significant amount of energy from the laser was absorbed, and the temperature of the tumor region increased significantly. As for the apoptosis ratio, it was observed that as the effective radius of the particle increased, the intensity of the laser with the maximum apoptosis ratio increased. This is due to the absorption coefficient not increasing at the same rate as the effective radius of the particle; thus, the effective absorption coefficient decreases, and a higher laser intensity is needed to induce apoptosis. Inversely, it was observed that as the volume fraction decreased, the laser intensity needed for apoptosis increased. This is because the volume fraction of the NPs is directly proportional to the absorbed light on the medium. Less NPs means less heat generated. In this work, the optimal conditions for PPT were confirmed through apoptosis ratio as well as the volume ratio needed to achieve the corresponding temperature needed to induce apoptosis [291].

Akyuz et al. conducted one of the few available numerical simulations on core–shell MPNPs [292]. They calculated heat transfer by using the COMSOL heat transfer module. At a 800 nm wavelength, the simulation showed the highest temperature increase was near the nanoparticle center, with an immediate decline in the surrounding medium. Nonetheless, the high-temperature regions were confined to the immediate surroundings of the NP. The iron oxide core/gold shell NP (100/25 nm) reported the highest temperature reaching 180 °C at its center, while decreasing below 50 °C within the first 1000 nm. When the medium was changed to simulate skin, the trends remained the same, with the exception that the temperatures at the center of the NPs remained high for long distances. This is likely due to the skin having lower thermal conductivity than water. The core–shell structure showed a maximum temperature of 300 °C, while showing slower heat loss at longer distances (50 °C at 3000 nm). Pure gold and iron oxide NPs, on the other hand, cool down faster than their core–shell counterparts [292].

## 7. Summary and Future Perspectives

We have summarized and discussed the synthesis, properties, and applications of MPNPs for cancer detection and treatment. MPNPs are typically prepared with the seed-mediated growth method, which can tune the core size, shell thickness and core and shell shape by manipulating synthetic parameters. The particles bear the magnetic properties of the core and optical properties of the metallic shell, which have been well investigated both experimentally and computationally. The strategies to functionalize the MPNPs range from physical adsorption to covalent binding with click chemistry, similar to metallic NPs. Due to their dual magnetic and optical properties, MPNPs have been widely used for cancer diagnosis and treatment based on different mechanisms, such as MRI for cancer imaging and MH for cancer therapy. Frequently, MPNPs have been used as a theragnostic agent, enabling simultaneous detection and treatment of cancer with a single nanoconstruct, due to the combined magnetic and optical properties of core–shell nanostructures.

Although MPNPs have proved their usefulness as theragnostic agents, their use does not come without challenges and limitations. Some nanomaterials like cobalt and silver can cause cytotoxicity due to the release of ions, which can trigger ROS reactions. The ROS act as a catalysis in Fenton-like reactions interacting with compounds like hydrogen peroxide (H_2_O_2_) and generate hydroxyl radicals. Metals like iron, silver, and copper can cycle between different oxidation states (Fenton reactions), allowing them to easily and rapidly donate or accept electrons, generating free radicals. These radicals prove to be detrimental to biomolecules causing stress and cellular damage to DNA, proteins, and lipids. When these ROS interact with biological components such as proteins, they attack the amino acids, particularly cysteine residues, involved in cellular pathways responsible for inflammation, cell growth, and stress response. They can also damage cells by exceeding the cell’s antioxidant capabilities, inducing damage to DNA by attacking and breaking the strands and causing base oxidation, disrupting cellular activity and contributing to aging, diseases, and cellular death (apoptosis and oncosis) [293,294,295].

In some cases, the immune system can be triggered, causing possible side effects or the explosion of the NPs from the body. When NPs enter the circulatory system, they can interact with immune cells due to their small size and surface properties. In contrast, immune cells will perceive the NPs as a foreign object which can lead to inflammatory response. This can be triggered through different pathways such as the NPs binding to pattern-recognition receptors (PRRs) or Toll-like receptors (TLRs) on immune cells. These receptors are responsible for recognizing pathogen-associated molecular patterns (PAMPs), which will induce an immune response by mistakenly signaling the presence of a hostile microbe. Another way is through opsonization, where NPs are absorbed onto the surface of red blood cells, forming a coating that the immune system can recognize as a foreign microbe, triggering phagocytosis [296,297,298,299,300]. Neutrophils are another immune cell that can act against NPs. It has been previously reported that neutrophils can trap NPs in their extracellular traps (NETs) by the activation of DNA receptors such as TLR9, which can be responsible for the recruitment of immune cells during an immune response [301].

The type of nanomaterial and the intended application need to be carefully considered for clinical applications. For instance, one of the most common ways to stabilize and allow a metal nanoparticle to be more biocompatible is by modifying its surface with polymers such as polyethylene glycol (PEG), chitosan, dextran, or polylactic acid. This will also impact the surface charge (zeta potential) of the NP, which can prevent aggregation by adjusting its ZP between −30 mV and +30 mV [201,302,303,304]. Furthermore, adding another metal like gold as an encapsulating shell can potentially improve biocompatibility by reducing the possibilities of ROS forming.

Another important aspect is understanding how NPs behave in the metabolic system. One of the main concerns is their toxicity due to lingering for long periods of time in our circulatory system and organs. This can potentially lead to immune responses and even organ damage. Some groups report the association of NPs in the differentiation of mesenchymal stem cells (MSCs). This disruption can be linked to various degenerative diseases. One case can be seen in the downregulation of the adipogenic differentiation of mesenchymal stem cells by interacting with the cell membrane and cytoplastic protein, inhibiting adipogenic differentiation of the MSCs [305]. NPs are mainly cleared from the body through the hepatic pathway where they are captured by the mononuclear phagocyte system, followed by accumulation on the spleen and liver, broken down by hepatocytes, and excreted through the urine or stool. Another way is through the renal pathway where the NPs are directly cleared by renal filtration and expelled through the urine [306].

In addition, many current synthetic methods use harmful reagents such as sodium borohydride, which can cause pulmonary edema at high levels of exposure through inhalation and, in the long term, cause damage to the central nervous system or react with water and moisture to produce hydrogen gas. It can also react with acids such as hydrochloric, sulfuric, or nitric acid to produce poisonous diborane gas. For clinical applications, there is a need to develop environmentally friendly MPNPs. However, only very few studies have made such efforts. Mirsadeghi et al. conducted an environmentally friendly synthesis of spherical IO-Au core–shell NPs using the aqueous extract of Carum carvi seeds [307]. The extract served as a reducing and capping agent during the synthesis. Natural honey has also been used to synthesize Fe_3_O_4_@Au NPs [308]. There is also a need for large-scale synthesis, which usually requires procedural automation. As discussed above, core–shell NP production typically involves a two-step process (core formation and shell deposition). Ahrberg et al. recently created an automated droplet reactor for the two-step synthesis of Fe_3_O_4_@Au NPs [249]. This method enables the ability to control particle size and morphology through precise synthesis conditions, promising for commercialization to make last scale MNPs for clinical applications. Other researchers, such as Pohling, are introducing new concepts through the creation of new MPNPs, such as the “Smart Nanorice”, that can be potentially used in biomedical applications [309].

As we progress through technological advancements, many oncology techniques will inevitably be affected by the driving force of modern medicine. We can expect AI to be incorporated into many current cancer diagnostic tools, such as mammography [310]. AI will not only help improve current methods, but it can also give rise to new oncology technologies to help further improve patients’ well-being. In our recent studies, we have shown that AI can expand the ability of the multiplexed SERS detection with IO-Au core–shell NPs to classify different subtypes of cancer cells in a mixed population [138]. We expect more AI-based studies will emerge in the next five years in nanotechnology-based cancer detection and treatment, including the use of MNPs, as they are a facile theragnostic nanoplatform. Recently, AI has been introduced in the design, synthesis and characterization of biomedical nanomaterials. This has led to increasing efficiency by reducing the reliance of trial-and-error-based experiments, designing efficient synthesis reaction parameters, and obtaining better characterization information [311].

As we continue to make technological breakthroughs, we will observe the emergence of new therapies and diagnostic tools. One type of technology that has been gaining the attention of many scientists is nanorobots. These devices are typically controlled through nanometric component assemblies that can even interact with the cellular membrane offering direct and specific interactions at the cellular level. Nanorobots are capable of carrying large volumes of anti-cancer drugs into the cancerous cells while avoiding healthy cells. Currently, nanorobots are designed to recognize 12 types of cancer cells. One of the challenges this technology faces is the environmental effects of the body. Sheer and viscous forces are present in our body, which need to be taken into consideration when creating the nanobots. Some possible strategies to overcome this include adjusting swimming patterns and navigation systems based on the area being used or introduced. Another way is their power sources. In cases where nanobots use enzyme reactions as their power source, this can lead to the inefficiency of the fuel process and low stability. Thus, more research needs to be conducted to explore more alternatives [312,313,314,315], such as the use of external forces such as magnetic fields, which can be constant and adjusted accordingly. Another possible way is a photon-excited power source, possibly by applying an NIR laser and thus stimulating the nanorobot

## Data Availability

No new data were generated in this study.
